# Distinct patterns of transcriptional and epigenetic alterations characterize acute and chronic kidney injury

**DOI:** 10.1038/s41598-018-35943-x

**Published:** 2018-12-14

**Authors:** Roya Sharifian, Daryl M. Okamura, Oleg Denisenko, Richard A. Zager, Ali Johnson, Sina A. Gharib, Karol Bomsztyk

**Affiliations:** 1 UW Medicine South Lake Union, University of Washington, Seattle, WA 98109 USA; 2 Seattle Children’s Research Institute, Center for Developmental Biology & Regenerative Medicine, University of Washington, Seattle, WA 98105 USA; 3 Computational Medicine Core, Center for Lung Biology, University of Washington, Seattle, WA 98109 USA; 4 The Fred Hutchinson Cancer Research Center Seattle, Seattle, WA 98109 USA

## Abstract

Acute kidney injury (AKI) and chronic kidney disease (CKD) are considered early and late phases of a pathologic continuum of interconnected disease states. Although changes in gene expression patterns have recently been elucidated for the transition of AKI to CKD, the epigenetic regulation of key kidney injury related genes remains poorly understood. We used multiplex RT-qPCR, ChIP-qPCR and integrative analysis to compare transcriptional and epigenetic changes at renal disease-associated genes across mouse AKI and CKD models. These studies showed that: (i) there are subsets of genes with distinct transcriptional and epigenetically profiles shared by AKI and CKD but also subsets that are specific to either the early or late stages of renal injury; (ii) differences in expression of a small number of genes is sufficient to distinguish AKI from CKD; (iii) transcription plays a key role in the upregulation of both AKI and CKD genes while post-transcriptional regulation appears to play a more significant role in decreased expression of both AKI and CKD genes; and (iv) subsets of transcriptionally upregulated genes share epigenetic similarities while downregulated genes do not. Collectively, our study suggests that identified common transcriptional and epigenetic profiles of kidney injury loci could be exploited for therapeutic targeting in AKI and CKD.

## Introduction

The alarming increase in the incidence of AKI reflects multiple factors such as aging of the population, drug toxicities, and complications from procedures^1^. Historically, it has been taught that patients who recovered from AKI did not have adverse long-term sequelae^2,3^. However, more recent studies have provided evidence that not only do patients who return to baseline function after significant AKI progress toward CKD, but those with CKD are much more susceptible to AKI and thus proceed on a downward spiral^2–5^.

Advances in our understanding of the cellular and molecular basis of AKI and CKD are demonstrating that these two syndromes are in fact pathophysiologically interconnected^1,6,7^. In AKI, the initial injury to tubular epithelial cells, peritubular capillary endothelial cells, resident phagocytic cells, and pericytes triggers inflammatory responses which include the release of chemokines/cytokines and the recruitment of leukocytes^8,9^. Tubular cells undergo apoptosis and necrosis followed by the clearing of cell debris by phagocytic tubular epithelial cells as well as macrophages^10,11^. Surviving tubular epithelial cells first dedifferentiate and proliferate, and then re-differentiate back to epithelial cells, and by doing so restore normal or near-normal tubule architecture^12–14^. In this “adaptive” AKI repair scenario the microvascular injury and inflammation also resolve^7,15^. However, during the recovery phase of AKI, maladaptive repair pathways can be triggered and escalate into progressive interstitial fibrosis^6,7^. Here, the dedifferentiated proliferating tubule cells undergo pathologic G2/M-arrest and become senescent, remain in an epithelial-mesenchymal transition (EMT) state, and secrete several profibrotic mediators such as TGFβ1^6,7,16,17^. The mechanistic factors that activate this trigger are largely unknown. Epigenetic regulation may play an important role in sustaining this activation. For example, aberrant promoter CpG island methylation can lead to transcriptional silencing of Rasal1 and promote renal fibrogenesis^18,19^.

Differential gene expression in part reflects both transcriptional and epigenetic changes. However, few studies have correlated gene expression with epigenetic changes in AKI and CKD models^20–22^ and no studies have been done across multiple models of acute and chronic injury. Understanding the role of epigenetic changes that are triggered during AKI and persist to drive CKD would open up potential avenues to mitigate renal injury. We used RT-qPCR and matrix chromatin immunoprecipitation (Matrix-ChIP) platforms^23,24^ in AKI and CKD mouse models to examine expression and epigenetic changes across a broad set of well-known kidney disease-associated genes.

## Results

To investigate the relationship between epigenetic and transcriptional changes in the context of progression from AKI to CKD, we used three established mouse models of AKI: i) unilateral ischemia reperfusion (IR) at 26 h, ii) LPS at 2 h, and iii) combination of IR and LPS; and two well-characterized models of CKD: i) unilateral IR at 24–26 days and ii) unilateral ureteral obstruction (UUO) at 14 days^25–29^. We first assessed mRNA expression levels using RT-qPCR of a large collection of genes previously implicated in either AKI or CKD, and from these genes selected a subset for detailed epigenetic analysis by ChIP-qPCR.

### AKI- and CKD-induced transcriptional changes

**Figure 1:**
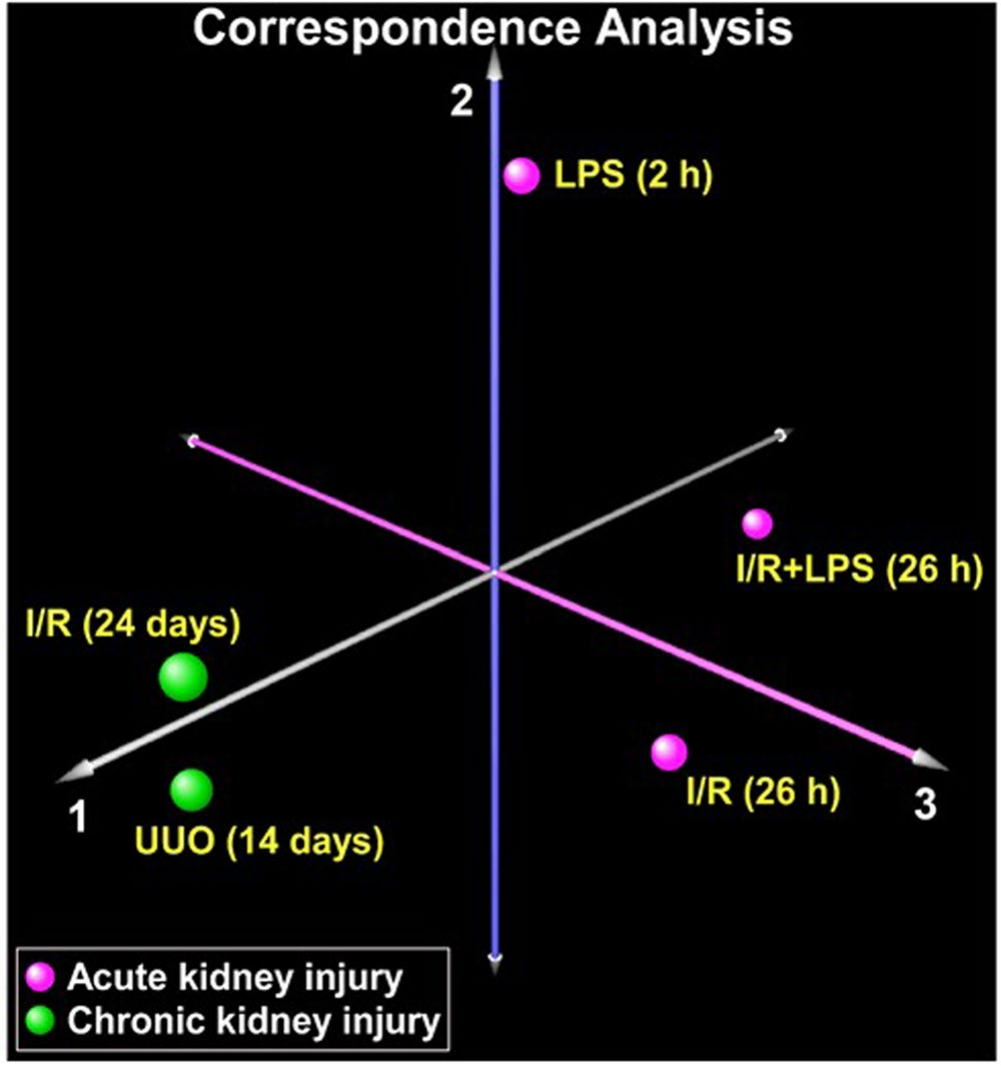
Correspondence analysis of mRNA expression variability across 120 candidate genes in acute kidney injury (AKI) and chronic kidney disease (CKD). Total RNA from injured kidneys and controls (contralateral) kidneys (n = 6 mice) was used in RT reactions with oligo-dT primers. cDNA was used in real time qPCR with gene specific primers (120 genes, Table S1, Supplement). mRNA level of a given gene in each sample was normalized to ribosomal RNA protein L32. For each experimental condition, the mean gene expression values from n = 6 mice were used for correspondence analysis. The analysis was based on assessing whether variability in gene expression across the experimental conditions distinguishes AKI models (magenta) from CKD models (green). Separation along each axis indicates differences in gene expression variability, with the first axis capturing the largest differences. As seen in the figure, AKI and CKD models segregated distinctly (axis 1), implying profound alterations in transcriptional signal between these two conditions. However, we also observed separation within the AKI models (axes 2 and 3) indicating gene expression variability between these acute renal injury conditions.

**Figure 2:**
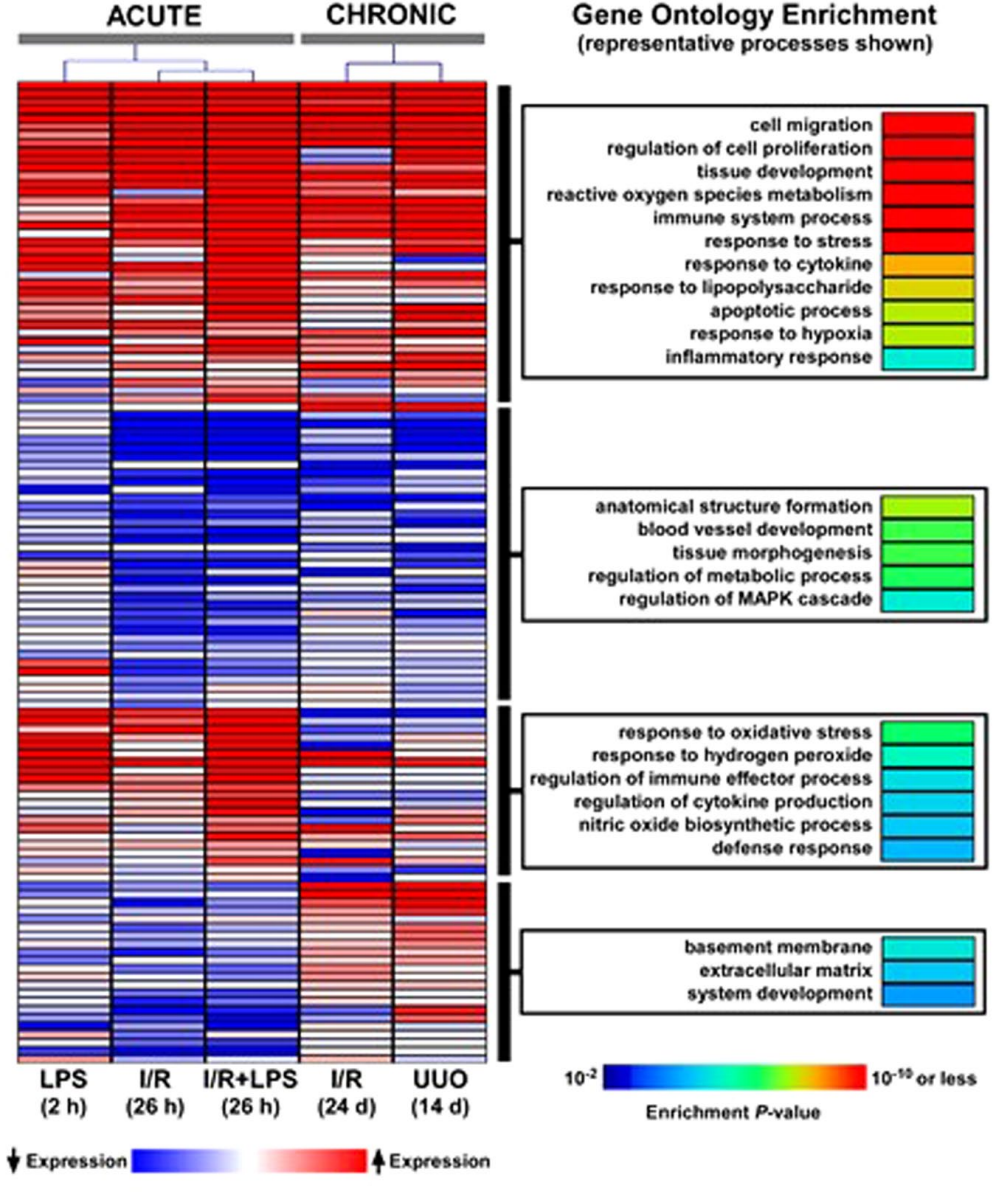
Correspondence analysis of mRNA expression variability across 120 candidate genes in acute kidney injury (AKI) and chronic kidney disease (CKD). Total RNA from injured kidneys and controls (contralateral) kidneys (n = 6 mice) was used in RT reactions with oligo-dT primers. cDNA was used in real time qPCR with gene specific primers (120 genes, Table S1, Supplement). mRNA level of a given gene in each sample was normalized to ribosomal RNA protein L32. For each experimental condition, the mean gene expression values from n = 6 mice were used for correspondence analysis. The analysis was based on assessing whether variability in gene expression across the experimental conditions distinguishes AKI models (magenta) from CKD models (green). Separation along each axis indicates differences in gene expression variability, with the first axis capturing the largest differences. As seen in the figure, AKI and CKD models segregated distinctly (axis 1), implying profound alterations in transcriptional signal between these two conditions. However, we also observed separation within the AKI models (axes 2 and 3) indicating gene expression variability between these acute renal injury conditions.

RT-qPCR^25^ was used to assess mRNA levels of a panel of 120 genes previously implicated in either AKI or CKD including pro-fibrotic mediators, Wnt/β-catenin pathway components, cytokines/chemokines, cell cycle, apoptosis, endothelial factors, hypoxia, components of extracellular matrix, and epigenetic mediators, (Tables S1, S3, S4 Supplement). We also included several well-studied kidney injury biomarkers. We assessed whether overall variability in gene expression across injury conditions can distinguish between the different models using correspondence analysis. We found that chronic injury transcript responses (IR 24–26 days and UUO 14 days) grouped together and clearly segregated from acute injury models (LPS 2 hrs, IR 26 hrs and I-R 26 hrs + LPS 2 hrs) (Fig. 1). In agreement with previous reports^25^, acute LPS and IR responses were also different from each other (Fig. 1). Analysis of these 120 candidate genes revealed four distinct expression patterns across injury models: genes that were either upregulated or downregulated in both AKI and CKD models, upregulated in AKI but not in CKD, and those that were upregulated in CKD but not in the AKI models (Figs 2 and S1). Although these 120 genes were not selected from an unbiased approach, we investigated whether the specific transcriptional patterns observed based on acute vs. chronic injury were associated with distinct functional groups using Gene Ontology (GO) analysis. Transcripts that were upregulated in both the AKI and CKD models were enriched in processes involved in cell migration and proliferation, tissue development, reactive oxygen metabolism, immune responses and other pathways known to be activated in renal injury. Transcripts that were downregulated in both AKI and CKD models were overrepresented in anatomical structure formation, blood vessel development, tissue morphogenesis and metabolic processes. The group of genes that were most highly upregulated in AKI but not in CKD included response to oxidative stress and hydrogen peroxide. Basement membrane and extracellular matrix processes topped the list of genes upregulated in CKD but not in AKI models consistent with evolving fibrogenesis as renal injury becomes more chronic. The GO analysis results are consistent with a recent transcriptome study^30^ and with cellular pathophysiology of AKI to CKD transition^1,6–8,15,30^.

**Figure 3:**
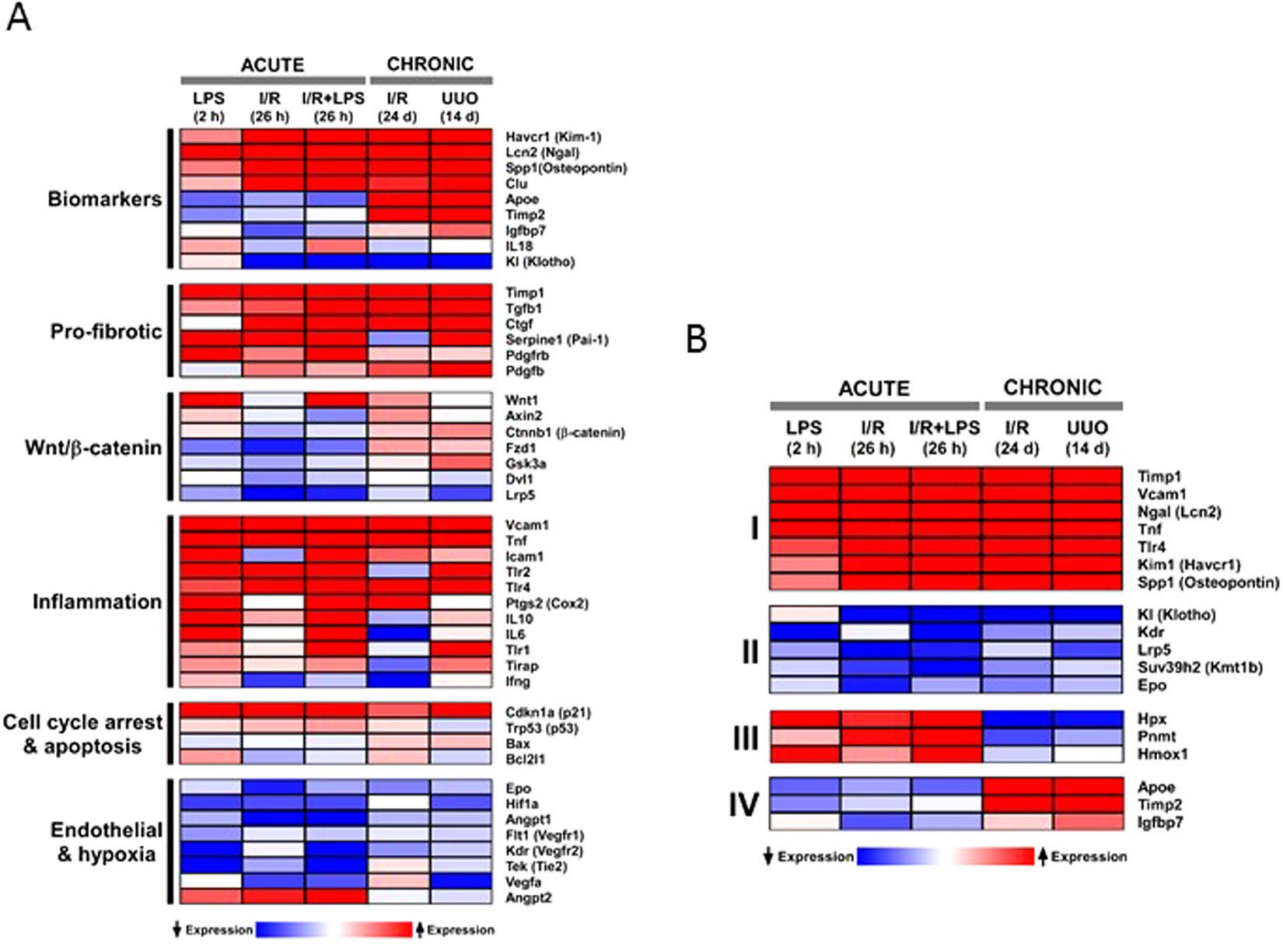
Heatmap representation of select candidate genes grouped based on their role in the pathogenesis of kidney injury. Database as in Fig. 2. (**A**) We chose to investigate a group of kidney injury-associated genes derived from the literature encompassing various putative roles in AKI and CKD. This analysis suggest the following. (i) mRNA levels of known biomarkers were increased in both AKI and CDK models (Kim-1, Ngal, osteopontin) while Apoe and Timp2 were highly elevated in CKD but not in AKI; (ii) several pro-fibrotic transcripts were upregulated across all AKI and CKD models; (iii) the overall magnitude of differential expression of Wnt/β-catanin pathway components was small; (iv) overall upregulation of inflammatory genes was higher in AKI than CKD; (v) except for p21 (which was upregulated across AKI and CKD) changes in cell cycle and apoptosis genes were small; (vi) generally there was downregulation of regulators of angiogenesis across AKI and CKD models; (**B**) Subset of genes with distinct expression patterns (see also Fig. 2) that were selected for detailed epigenetic analyses. There are shared transcriptional changes across AKI and CKD models with some exceptions (e.g. Apoe and Timp2) that could differentiate late from early injury.

Several biomarkers of AKI have been validated and the mechanistic basis for their production are being defined^31–33^. Transcripts encoding AKI biomarkers including Kim-1 (Havcr1), Ngal (Lcn2), Spp1 (osteopontin) and Clu (clusterin) were increased across all AKI and CKD models (Figs 2 and 3, S1). Urinary TIMP-2 protein (tissue inhibitor of metalloproteinases 2) is a biomarker of renal injury^31,34^ and together with IGFBP7 (insulin-like growth factor binding protein 7) have been shown to be associated with adverse long-term outcomes in patients with AKI^34,35^. In contrast to Kim-1 and Ngal, Timp-2 and Igfbp7^34,35^ were upregulated in the CKD but not in the AKI models. As serum creatinine is a poor marker of kidney function especially in children and the elderly, there is an unmet need to have additional biomarkers that define the AKI to CKD transition^5^. We identified several other genes that clustered with Timp-2 and Igfbp7 and were upregulated in CKD but not in AKI models, among them Apoe (apolipoprotein E) showed the largest difference between the two renal injury states (Fig. 3). Although little is known mechanistically about Apoe in renal injury, genetic variation in this gene has been linked to CKD progression^36^. The differences between Apoe mRNA levels in CKD compared to AKI models (also see^30^) suggest a potential value to explore urinary levels of APOE, together with TIMP-2, IGFBP7 and other proteins, to develop composite biomarkers of AKI-to-CKD progression.

Several pro-fibrotic-related transcripts were upregulated across all AKI and CKD models including Tgf-β, Ctgf, Pai-1, Pdgfβ and Pdgfr (Figs 3A and S1). This observation confirms previous reports that upregulation of these genes plays a role in the pro-fibrotic events, and our findings suggest that these effects start early in the injury process and persist.

Activated Wnt/β-catenin signaling pathway plays a key role in AKI to CKD progression, at least in part, by driving interstitial fibrosis^37–39^. We examined expression of several components of the Wnt pathway including Wnt1 (ligand), Fzd1 (receptor for Wnt) Ctnnb1 (β-catenin, transcription coactivator), Gsk3a (kinase), Axin2 (negative regulator), Dvl1 (DSH), and Lrp5 (co-receptor)^40^. The overall magnitude of differential expression of genes encoding components the Wnt/β-catenin pathway was smaller (compared to pro-fibrotic or biomarker genes) (Fig. 3A) suggesting that control of these pathways in kidney injury is predominantly at mRNA translation, protein modification and degradation steps^40^.

Sterile inflammatory response within the kidney is a hallmark of both AKI and CKD^5,12^. Several transcripts encoding known inflammatory mediators are upregulated in AKI (Tnf, Ccl2 (Mcp-1), Tlr2, Trl4, IL6, IL10, IL18, Cox2, Icam1 and Vcam1) (Fig. S1), but the expression levels of some of these genes decreased in the CKD phase (Fig. 3A). Overall, this upregulated set of genes indicates a higher inflammatory state in AKI compared to CKD.

Genes important in cell death and proliferation are induced following acute kidney injury and with the onset of CKD^30^. Increased expression of the cyclin-dependent kinase inhibitor p21 in AKI plays a key role in epithelial cell cycle arrest which appears to be beneficial in the acute phase^41–43^ but in the progression of AKI to CKD can lead to fibrosis^43,44^. p53 is a major regulator of cell death and its inhibition results in markedly reduced proximal tubular injury^45,46^. We have found that p21 mRNA expression was strongly upregulated in both the AKI and CKD models, while p53 transcript increases were small or unchanged (CKD UUO) (Figs 3A and S1). p53 regulates transcription and/or activity of several apoptosis factors including the pro-apoptopic Bax and anti-apoptopic Bcl1L2(Bcl-x). Bcl2 family of proteins have been implicated renal injury^47,48^. Given that changes of mRNAs encoding Bax and Bcl1L2/Bcl-x were relatively small suggest that mRNA translation and/or post-translational regulation of these factors maybe playing a more significant role in renal injury.

Tubulointerstitial hypoxia is a major contributor to the pathogenesis of AKI-to-CKD progression^5,6,20,49–51^. The mechanisms of capillary loss involve a host of endothelial and hypoxia-responsive factors^52^. The endothelial Ang-Tie-2 and VEGF-VEGFR signaling systems control angiogenesis and microvascular integrity^53,54^ and their downregulation (for example in sepsis) causes endothelial dysfunction and microvascular leak^55^. mRNA expression of several components of these signaling pathways (Angpt1, Flt1, Kdr, and Tek(Tie-2)) were downregulated across all models except for Angpt2 which was upregulated (Figs 3A and S1). Angpt1 and Angpt2 effects are typically antagonistic^56^ so their difference in mRNA expression is consistent with their opposing roles. The downregulation of key regulators of angiogenesis confirms the ongoing capillary dysfunction in the transition of acute to chronic kidney disease^57^.

### Epigenetic changes in AKI and CKD

Transcript analysis identified subsets of genes that were either induced or repressed across the AKI and CKD models (Figs 2 and 3, S1). Although epigenetic changes such as DNA methylation are associated with CKD^58^, less is known about the correlation between histone modifications and the transcriptome during AKI and CKD. For our epigenetic studies, we selected representative candidate genes from each of the four identified expression patterns: i) upregulated in AKI and CKD models, Kim-1, Ngal, Tnf, Tlr4, Timp1 and Spp1(osteopontin); ii) downregulated in AKI and CKD, Klotho (KI), Lrp5, Kmt1b (Suv39h2), Kdr, and Epo; iii) upregulated in AKI but not in CKD, Hpx, and Pnmt, Hmox1; and iv) upregulated in CKD but not in AKI, Apoe, Timp2 and Igfbp7 (Fig. 3B). The choice of genes was driven by potential translational application in clinical settings^5,34,59,60^. We used the following ChIP-validated antibodies in Matrix ChIP: RNA Polymerase II CTD (Pol II CTD) as a measure of transcription levels; H3K27Ac, H3K4m1, H3K4m2, H3K4m3 permissive marks; and H3K27m3, H3K9m2, H3K9m3 repressive marks (Table S4)^25,26,55,61^. Across all models we found that within a given gene subset, the permissive marks H3K27Ac and H3K4m2, and the repressive H3K9m2 marks showed the most consistent changes with the transcript levels. In contrast, the permissive H3K4m1, H3K4m2 and repressive marks, H3K27m3 and H3K9m3 were less correlated with transcript levels and exhibited greater heterogeneity. We therefore have focused our discussion on H3K27Ac, H3K4m2 and H3K9m2 modifications. All ChIP data for each gene are shown in the Supplementary material (Figs S2–S20).

#### Genes upregulated in AKI and CKD

**Figure 4:**
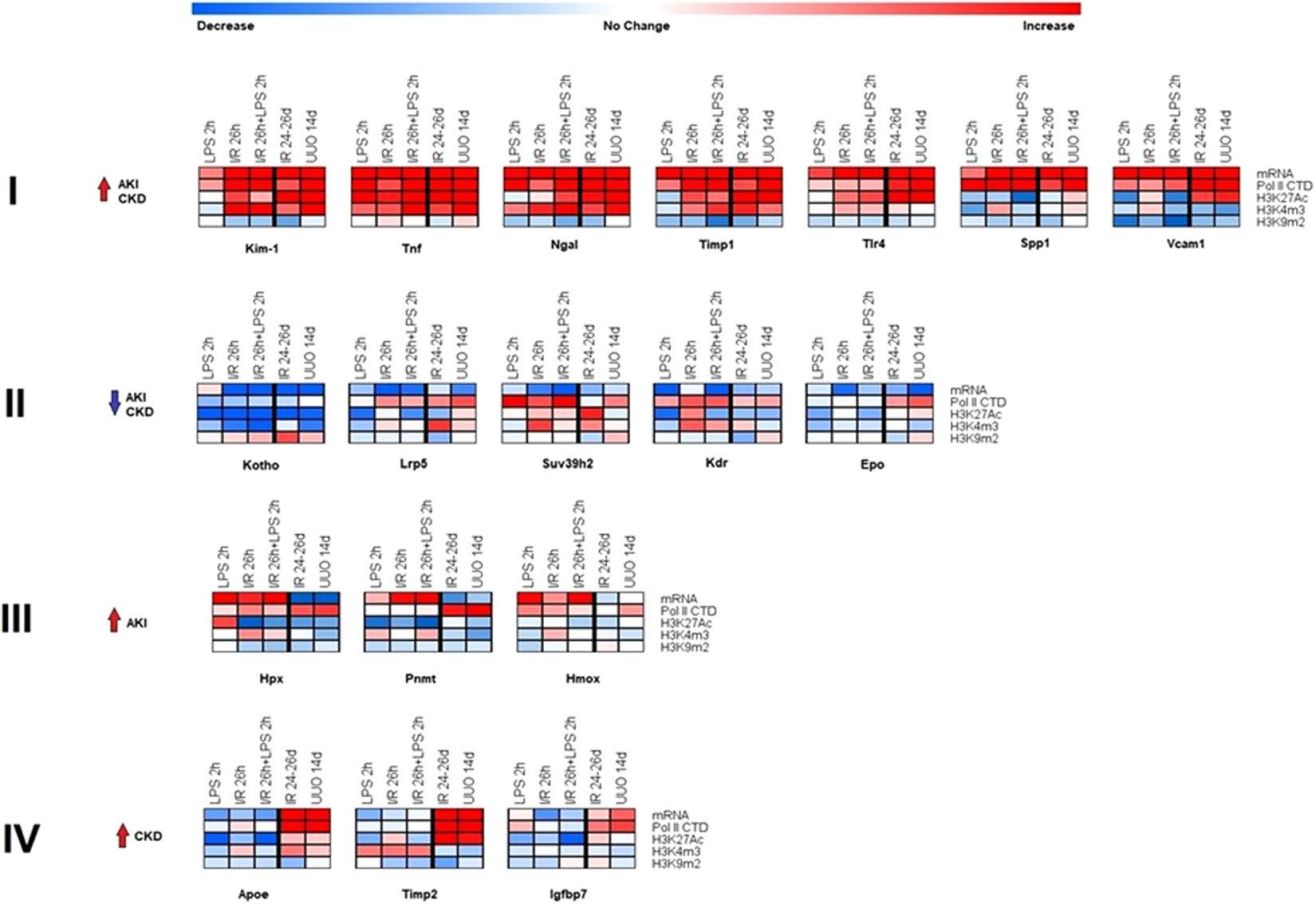
Integrated transcriptional and epigenetic analysis of selected sets of renal injury genes from AKI and CKD models. *RT-qPCR RNA analysis*. Total RNA from injured kidneys and controls kidneys (n = 6 mice for each model) was used in RT reactions with oligo-dT primers. cDNA was used in real time qPCR with gene specific primers (same as Figs 1–3). Transcript levels were normalized to ribosomal protein mRNA L32 (same as Figs 1–3) *ChIP-qPCR analysis*. Tissue fragments from injured and control (contralateral) kidneys (n = 6 mice for each injury model) were crosslinked and sonicated. Sheared chromatin was analyzed in Matrix ChIP-qPCR using antibodies to RNA polymerase II (Pol II) and antibodies to permissive (H3K27Ac, H3K4m3) and repressive (H3K9m2) marks. ChIP signals were normalized to input. Data represents log2 transformed ratios of means from injured kidneys over controls. Shown are genes whose expression was: (I) upregulated in both AKI and CKD; there was agreement between the increased in mRNA, Pol II and the permissive H3K27Ac levels. For most of these genes there was also increased in permissive H3K4m3 modification but decreased H3K9m3 repressive mark; (II) downregulated in both AKI and CKD;Pol II levels were either increased or in case of Klotho slightly decreased with decreased permissive but increased repressive modifications at this gene. (III) upregulated in AKI but not in CKD; Pol II levels in AKI were only moderately elevated and the epigenetic changes were small and (IV) upregulated in CKD but not in AKI; there was corresponding increase in Pol II and permissive H3K27Ac.

**Figure 5:**
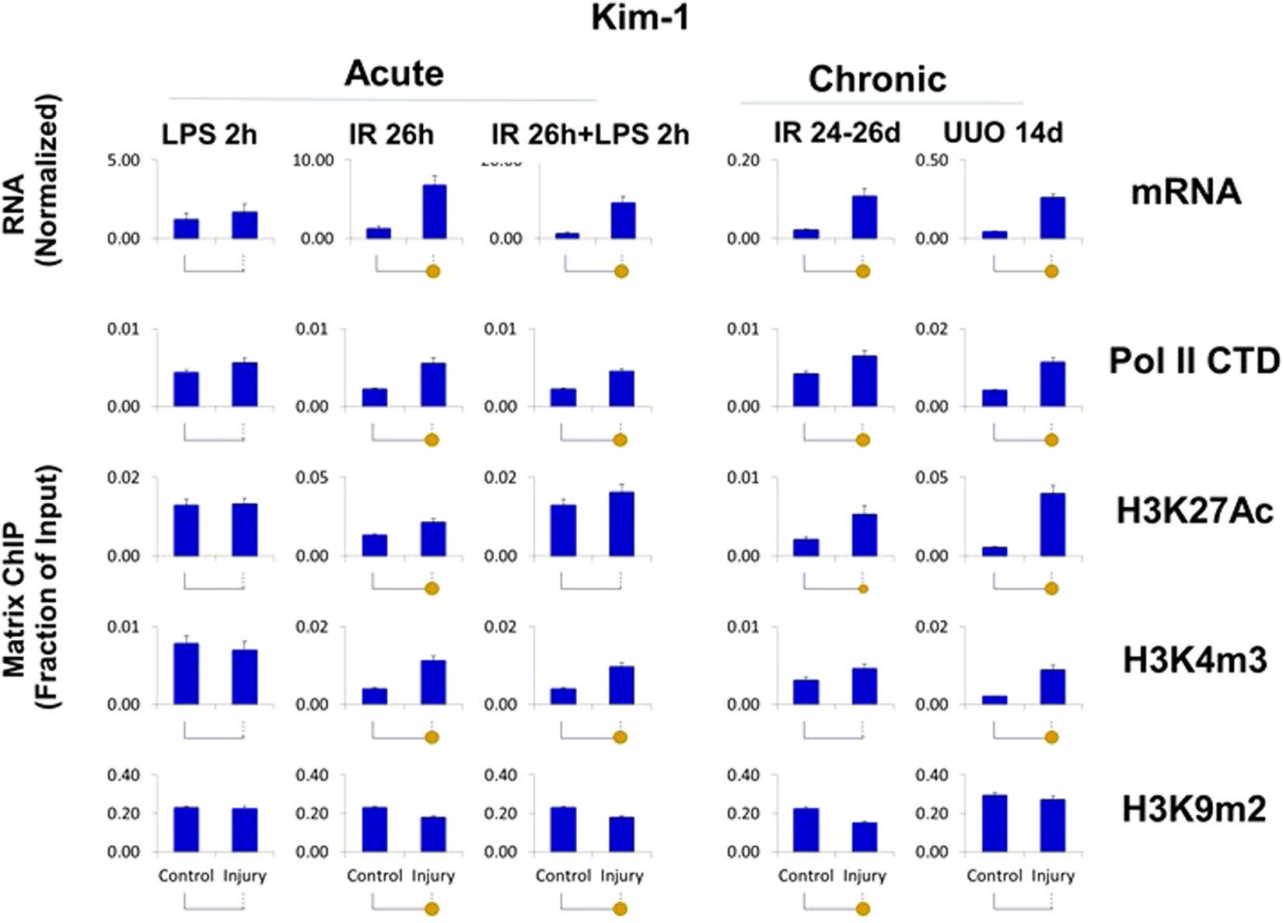
Transcriptional and epigenetic analysis of Kim-1 (Havcr1) gene in AKI and CKD models. RT-qPCR RNA and ChIP-qPCR analysis (see Fig. 4). Kim-1 transcript levels (mRNA) were normalized to L32 mRNA. Matrix ChIP-qPCR analysis of RNA polymerase II (Pol II), and permissive (H3K27Ac, H3K4m3) and repressive (H3K9m2) marks (n = 6 mice from each group) Small solid circle, p < 0.05, large circle, p < 0.01, and no circle, not statistically significant. Kim-1 transcript levels were upregulated in all model expect for LPS. mRNA levels were matched by increased in Pol II and permissive modifications and reduced repressive marks suggesting transcriptional and epigenetic control of gene expression.

**Figure 6:**
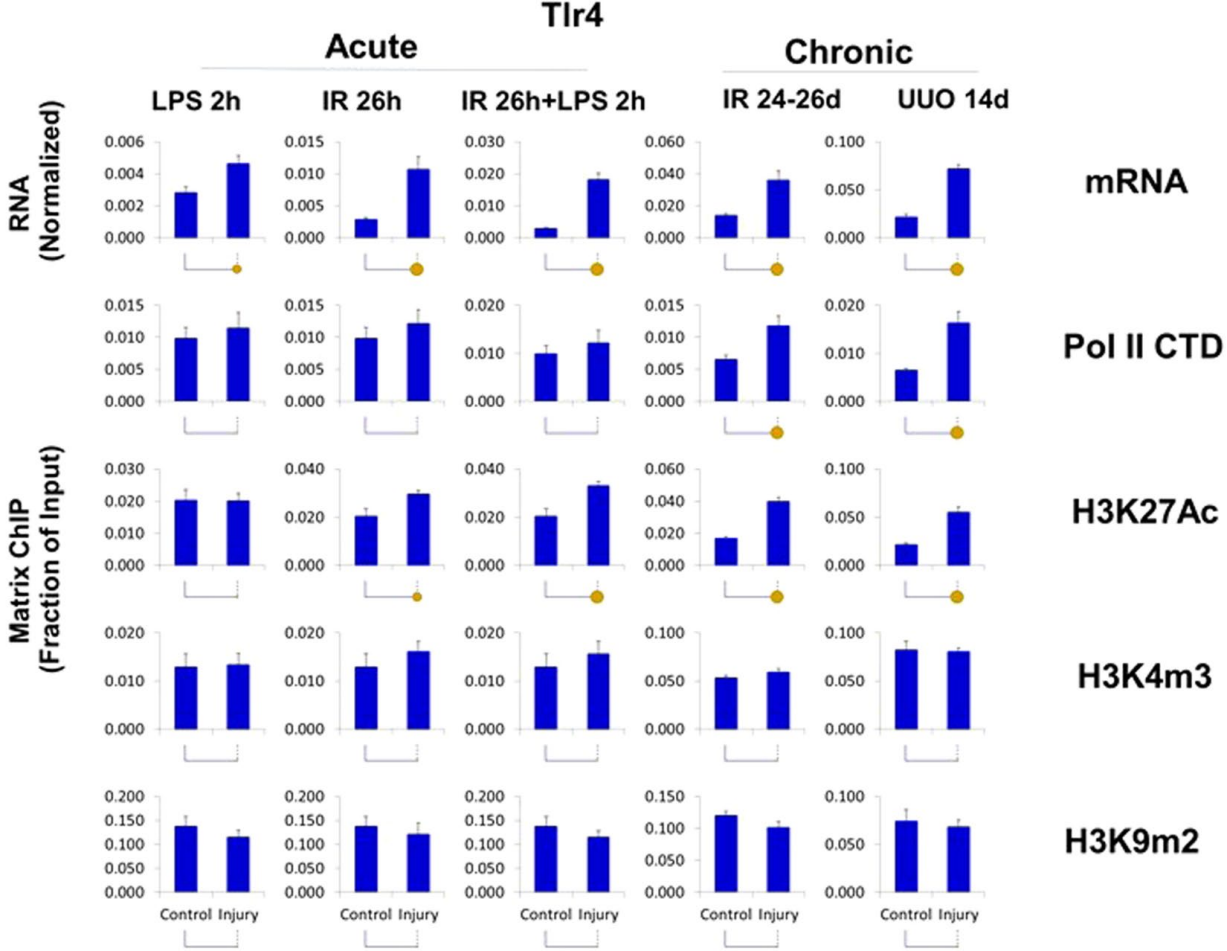
Transcriptional and epigenetic analysis of Tlr4 gene in AKI and CKD models. RT-qPCR RNA and ChIP-qPCR analysis (see Fig. 4). Tlr4 transcript levels (mRNA) were normalized to L32 mRNA. Matrix ChIP-qPCR analysis of RNA polymerase II (Pol II), and permissive (H3K27Ac, H3K4m3) and repressive (H3K9m2) marks (n = 6 mice from each group). Small solid circle, p < 0.05, large circle, p < 0.01, and no circle, not statistically significant. The upregulated Tlr4 mRNA was matched by increased in Pol II levels in CKD and less so in AKI, where in the latter post-transcriptional processes maybe also playing a role.

The upregulated transcripts (Kim-1, Ngal, Timp1, Tlr4, Tnf, Spp1, and Vcam1) were associated with higher levels of Pol II at the genes (Figs 4–6, S2–S9) suggesting that, at least in part, increased expression of these genes is transcriptionally mediated. Although all seven genes were upregulated, there were two strikingly different epigenetic subsets of genes. In one group, exemplified by Kim-1 (which also included Ngal, Timp1, Tlr4 and Tnf), upregulation of transcript and Pol II levels correlated with chromatin permissive structure as determined by increased histone acetylation and methylation marks H3K27Ac and H3K4m3, respectively (Figs 4–6, S2–S6, S9). In contrast, in the second group, there was not such correlation, i.e. transcriptional upregulation was not associated with increased H3K27Ac (Spp1) or H3K3m3 (Spp1, Vcam1) marks (Figs 4 and S2, 7, 8). All genes in this set (with the exception of Tnf) showed AKI and CKD-induced decreases in the repressive H3K9m2 modification, a change correlated with their upregulation.

#### Genes downregulated in AKI and CKD

**Figure 7:**
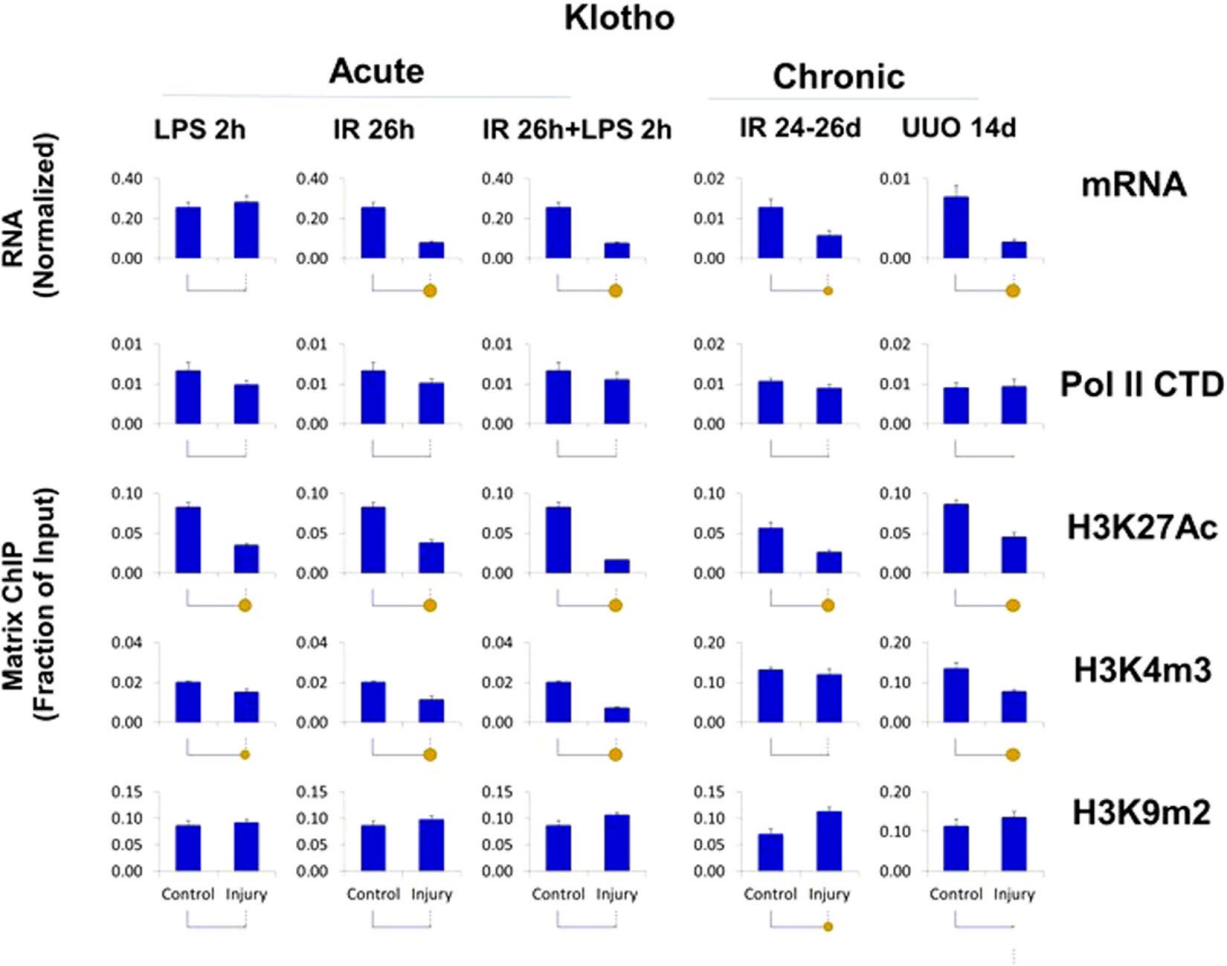
Transcriptional and epigenetic analysis of Klotho (KI) gene in AKI and CKD models. RT-qPCR RNA and ChIP-qPCR analysis (see Fig. 4). Klotho transcript levels (mRNA) were normalized to L32 mRNA. Matrix ChIP-qPCR analysis RNA polymerase II (Pol II), and permissive (H3K27Ac, H3K4m3) and repressive (H3K9m2) marks (n = 6 mice from each group). Small solid circle, p < 0.05, large circle, p < 0.01, and no circle, not statistically significant. The downregulated Klotho transcripts were not matched by Pol II levels suggesting that post-transcriptional mechanisms are playing a role (e.g. splicing, message stability). Here, the epigenetic changes that correspond to mRNA levels could be playing a role.

The set of mRNAs downregulated in both AKI and CKD models (Klotho, Lrp5, Suv39h2, Kdr and Epo) (Figs 4, 7 and S10–14) were not associated with matched decrease in Pol II levels suggesting that reduced message stability, rather than transcriptional suppression, play a more significant role in their lower expression. This was illustrated by Klotho (Figs 4, 7, S2 and S10), an increasingly important AKI and CKD biomarker, whose decreased levels are associated with worse renal outcomes^60,62^. On the one hand, decreased Klotho mRNA levels mirrored lower H3K27Ac and H3K4m3 marks while on the other there was a trend towards increased H3K9m2 levels. Other genes in this group, Lrp5 and Kdr, also exhibited decreased H3K27Ac but increased Pol II levels (Figs 4, S2, S11 and S13), again suggesting that their downregulation was meditated post-transcriptionally. In all these cases decreased transcription could also have been a contributing factor in their downregulation.

#### Genes upregulated in AKI only

The common feature of genes in this set (Hpx, Pnmt and Hmox) (Figs 4, S2 and 15–S17) was the fact that the large AKI-induced increases in their transcript levels occurred with little or no change in Pol II density at these genes. Further, in both CKD models there was no increase in the mRNA levels in the injured kidney even though the Pol II levels at these genes were higher. These traits are shown for Hpx, Pnmt and Hmox in Figs S15–S17. It has previously been shown that AKI-induced Hpx expression is due, at least in part, to increased mRNA stability^63^. These results imply that at the time points examined epigenetic alterations are not playing a predominant role in regulating mRNA levels of these three genes in response to kidney injury. Alternatively, the epigenetic alterations occurred in a subpopulation of cells and therefore were not detectable in whole kidney samples.

#### Genes upregulated in CKD only

**Figure 8:**
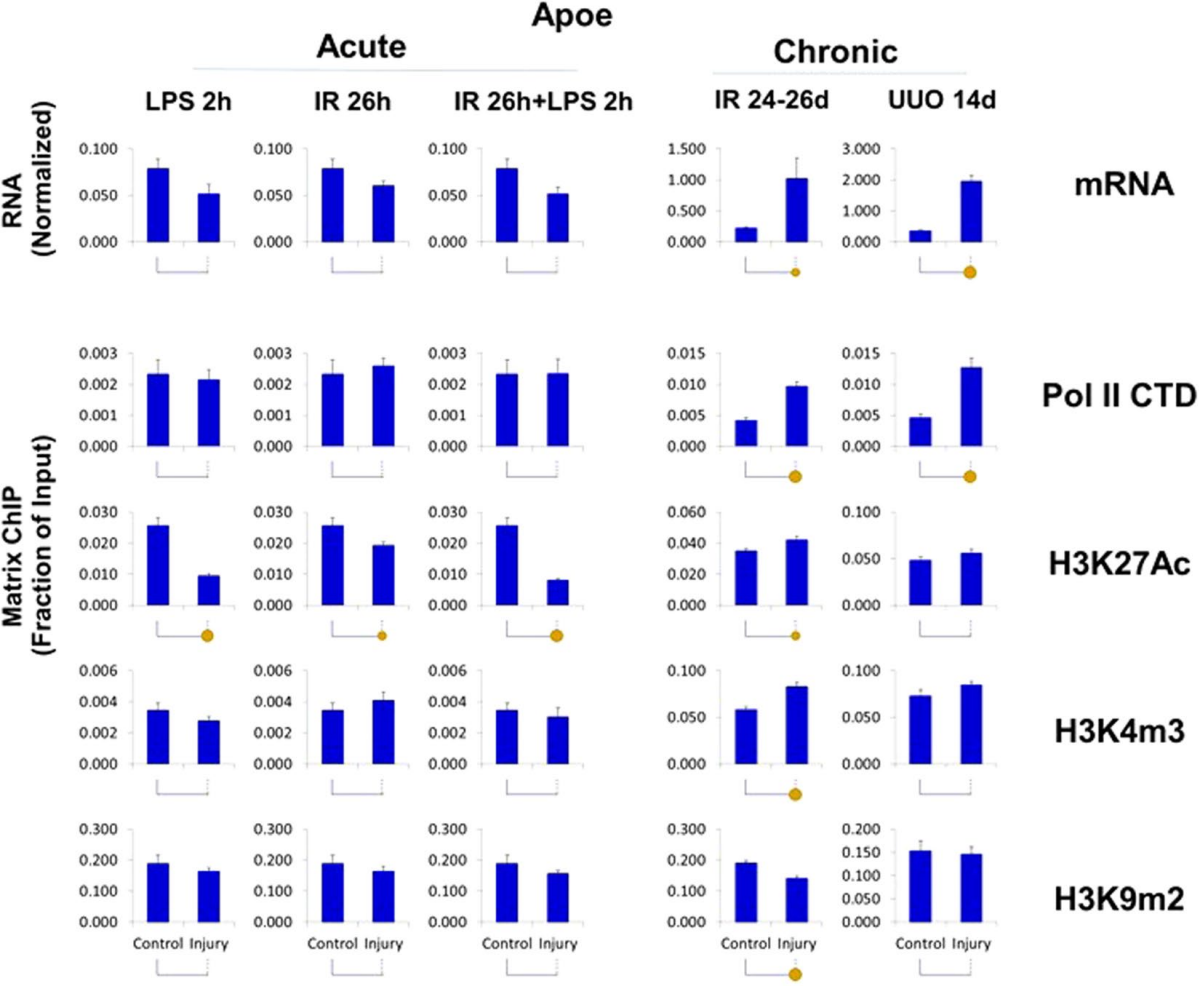
Transcriptional and epigenetic analysis of Apoe gene in AKI and CKD models. RT-qPCR RNA and ChIP-qPCR analysis (see Fig. 4). Apoe transcript levels (mRNA) were normalized to L32 mRNA Matrix ChIP-qPCR analysis of RNA polymerase II (Pol II), and permissive (H3K27Ac, H3K4m3) and repressive (H3K9m2) marks (n = 6 mice from each group). Small solid circle, p < 0.05, large circle, p < 0.01, and no circle, not statistically significant. Apoe was upregulated in CKD but not AKI. The corresponding increase in Pol II and permissive marks and decreased repressive mark indicate that the upregulation is transcriptionally and epigenetically mediated.

For ChIP analysis we selected three genes in this set, Apoe, Timp2 and Igfbp7. For all three genes, the CKD induced mRNA increases were matched by elevated Pol II and the permissive mark H3K27Ac levels at these loci (Figs 4 and S2). This is illustrated for Apoe in Fig. 8 (see also Figs 4, S2 and S18) and for Timp2 and Igfbp7 in Figs 4, S2 and S19–S20. The response of the Igfbp7 gene was significantly smaller than for the other two genes. Overall, these results suggest that the increased expression of this set of genes in CKD was predominantly transcriptionally mediated and that the increase in the permissive mark H3K27Ac could have contributed to this effect.

### Comparison of transcriptional and epigenetic profiles in sham, contralateral and unilaterally injured kidneys in CKD models

**Figure 9:**
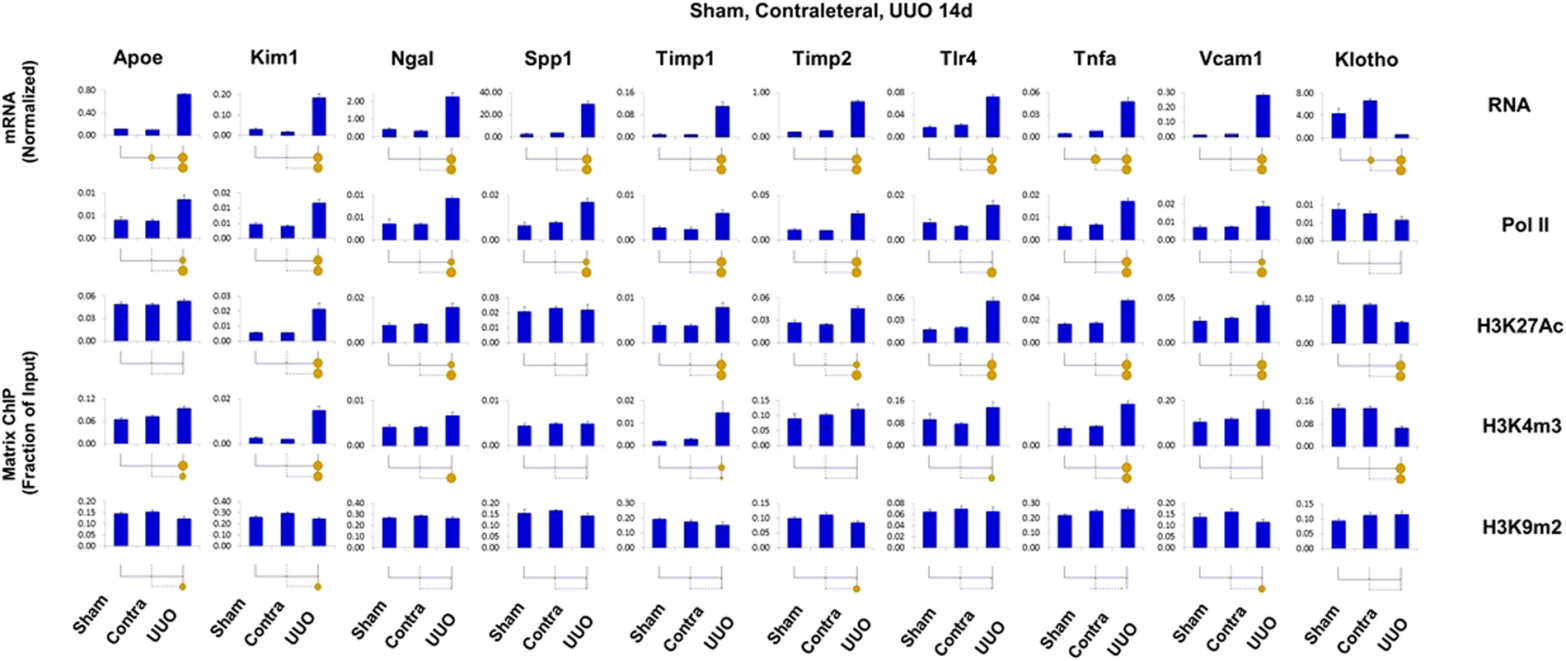
Transcriptional and epigenetic analysis in sham, contralateral and UUO CKD model. Total RNA and sheared chromatin was prepared from UUO, contralateral (Contra) and sham (Sham) kidneys 14 days after surgeries. Sham UUO surgeries were performed in the same manner as the disease model surgeries but without tying the ureter. RT-qPCR RNA and ChIP-qPCR analysis (see Fig. 4). Transcript levels (mRNA) were normalized to L32 mRNA. Matrix ChIP-qPCR analysis of RNA polymerase II (Pol II), and permissive (H3K27Ac, H3K4m3) and repressive (H3K9m2) marks (n = 6 mice from each group). Small solid circle, p < 0.05, large circle, p < 0.01, and no circle, not statistically significant. The results demonstrate that transcript and epigenetic differences between contralateral and sham kidneys are either small or not detectable. Thus, the contralateral kidneys are appropriate internal controls for this chronic UUO model.

**Figure 10:**
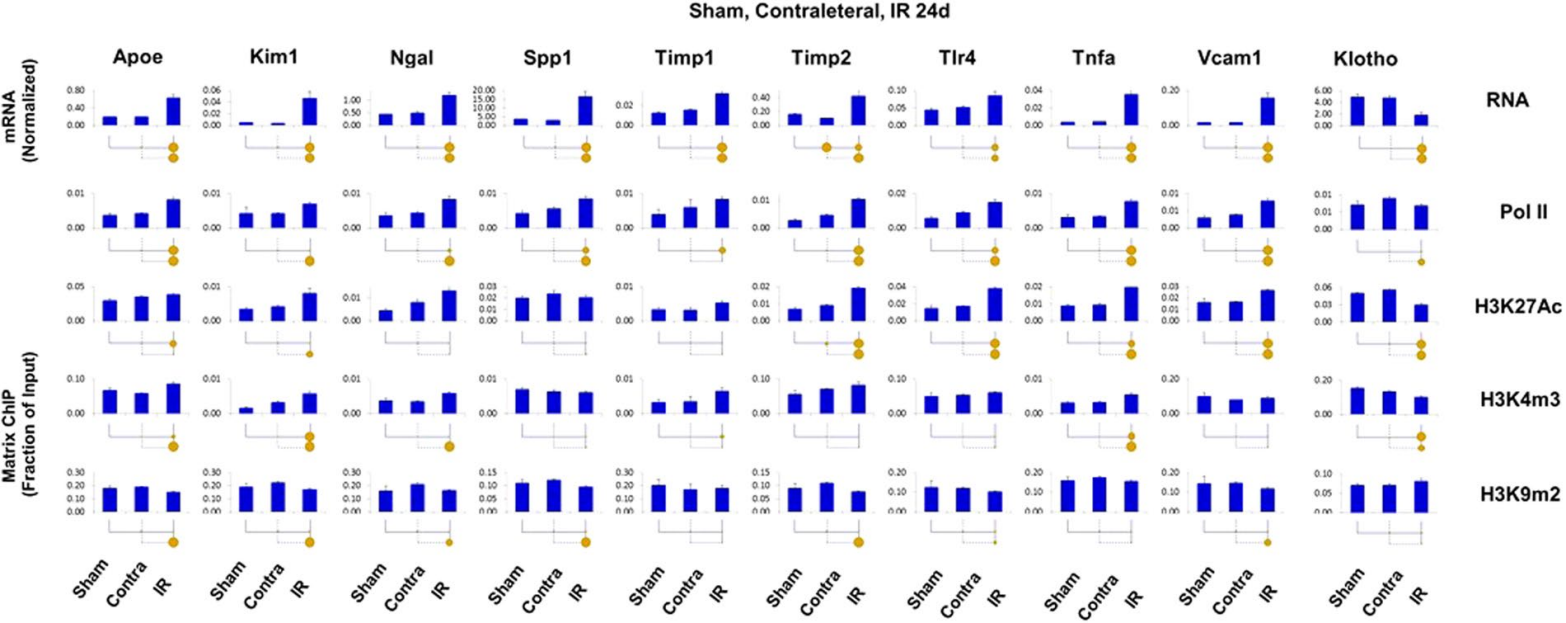
Transcriptional and epigenetic analysis in sham, contralateral and IR CKD model. Total RNA and sheared chromatin was prepared from UUO, contralateral (Contra) and sham (Sham) kidneys 24 days after surgeries. Sham IR surgeries were performed in the same manner as the disease model surgeries but without clamping the vascular pedicle,. RT-qPCR RNA and ChIP-qPCR analysis (see Fig. 4). Transcript levels (mRNA) were normalized to L32 mRNA. Matrix ChIP-qPCR analysis of RNA polymerase II (Pol II), and permissive (H3K27Ac, H3K4m3) and repressive (H3K9m2) marks (n = 6 mice from each group). Small solid circle, p < 0.05, large circle, p < 0.01, and no circle, not statistically significant. The results demonstrate that transcript and epigenetic the differences between contralateral and sham kidneys are either small or not detectable. Thus, the contralateral kidneys are appropriate internal controls for this chronic IR model.

The use of contralateral kidneys in unilateral renal injury models has traditionally been used to decrease complexity and cost of experiments and to control for variability between animals. For example, the use of contralateral kidneys in unilateral AKI models has been routinely been used including epigenetic studies^25,64–68^. Still, in unilateral CKD models it is possible that the contralateral kidneys may undergo epigenetic changes over time so that using them as internal controls for chronic renal injury could confound data interpretation. To address this issue we carried out a series of experiments in UUO and IR CKD models where we profiled transcriptional and epigenetic renal profiles in contralateral, unilaterally injured kidneys and kidneys from sham operated mice. For these CKD studies, we chose genes whose transcripts and epigenetic modifications changed the most. This parallel analysis revealed that the differences between sham and contralateral kidneys were either small or not detectable (Figs 9 and 10) confirming above conclusions based on using contralateral kidneys as internal controls (Figs 5–8). This is in agreement with other CKD studies that showed no differences in mRNA expressions in sham and contralateral kidneys^69–71^, These transcriptional and epigenetic measurements demonstrate that the use of contralateral kidneys as an internal controls in unilateral CKD models is appropriate allowing to use fewer mice, expediting such studies and saving costs.

## Discussion

Our study is the first to simultaneously compare the transcriptional and epigenetic profiles of a panel of 120 genes previously implicated in kidney disease across acute and chronic models of renal injury (Fig. 4). We identified distinct subsets of transcription and epigenetically defined genes that are shared by AKI and CKD but also ones that are specific to either the early or late stages of renal injury. We found that expression of more than half of the genes was similarly altered in the AKI and CKD models with the remainder displaying distinct transcriptional patterns based on the temporal duration of injury (Fig. 2). Similarly, a recent unbiased RNA-seq approach also demonstrated that subsets of genes are altered at the onset and those that change expression days later persist in the progression from AKI to CKD^30^, suggesting that different epigenetic pathways are involved in gene expression alterations^2,3^.

Why some kidney injury transcriptional changes persist while others resolve or arise later in the course of renal injury is not known. We assessed transcription (Pol II levels) and histone modification profiles as one way to address this question. We found that increased expression of genes in both AKI and CKD models, is primarily transcriptionally mediated while post-transcriptional processes may play a more significant role in gene downregulation in the setting of kidney injury (e.g. Klotho, Figs 7 and S10). Although post-transcriptional regulation of message stability in renal injury has been known for more than a decade^63,72,73^ it is a critical area for investigation. Along these lines, it has recently been shown that renal injury-induced Klotho transcript downregulation reflects alternative splicing that leads to mRNA degradation by nonsense-mediated transcript decay^74^. Histone modifications play a role in alternative splicing^75^. Thus our results suggest that the decrease in Klotho message levels in response to renal injury could reflect, at least in part, epigenetic changes at this locus. Recognizing the key role of microRNAs (miRNAs) and other non-coding RNAs in the regulation of message stability is revealing new mechanisms of post-transcriptional processes in the pathophysiology of kidney disease progression^76^. Most notably, global reduction of miRNAs by conditional Dicer deletion in proximal renal tubules protects from ischemic AKI^77^. Thus, it seems likely that miRNAs play a key role in the AKI-to-CKD continuum. The challenge for future studies will be the identification of renal injury-induced specific miRNAs that participate in the increased degradation of transcripts relevant to AKI –to-CKD transition.

For the epigenetic studies, we focused on a set of histone marks that are among the best studied^61^. There was a correlation between AKI and CKD-induced changes in gene expression and histone marks for some AKI and CKD genes (e.g., Kim-1, Ngal, Timp1, Tlr4, Tnf and Klotho) (Figs 4 and S1). For genes that were upregulated in CKD only (e.g., Apoe and Timp2) there was an increase in H3K27Ac permissive modification which could have played a role in the AKI-to-CKD transition. For other genes, even those that were potently affected by renal injury (e.g. Spp1, Figs 3 and 4, S7), there were no consistent epigenetic changes. There are more than one hundred different chromatin modifications and the lack of coherent epigenetic changes may simply reflect our focus on a small group of modifications. Further, chromatin modifications have roles other than control of gene transcription including mRNA processing (e.g., splicing), maintaining nuclear architecture, priming, and inheritance^61,75,78,79^. We expect that further advances in high throughput epigenetic technologies will permit simultaneous profiling of many more epigenetic changes, allowing us to more comprehensively define the relationships between chromatin, transcription and post-transcriptional processes in renal injury.

This study revealed unique transcriptional and epigenetic responses to renal injury. Development of small molecules that target epigenetic enzymes and factors is one of the fastest growing therapeutic areas^61,80–82^. This trajectory reflects the fact that most of gene expression alterations are mediated by reversible epigenetic changes. Recent studies show that epigenetic drugs are gene selective which, likely, is dictated by unique local chromatin landscape^83^. Further, when used in combination epigenetic agents act synergistically which further underscores their potential to target specific groups of genes to mitigate progression of AKI to CKD^61,82–84^ while minimizing off target effects. For example, repletion of renal injury-induced Klotho deficiency mitigates renal injury^85^. Thus, it is formally possible that co-administration of selective and synergistically acting HDAC^86^ and lysine demethylase (KDM5B)^87^ inhibitors could reverse decreased H3K27Ac and H3K4m3, respectively (Fig. 7), and by doing so, restore Klotho expression and lessen or prevent chronic renal injury. Thus, this study provides a useful compendium of candidate factors for selective epigenetic interventions.

There are several limitations in our study. qPCR-based assays limited the breadth of this study to a relatively small set of genes and epigenetic modifications in five AKI and CKD models. Less biased approaches using next generation sequencing (NGS) technologies (e.g. ChIP-seq) should further advance our understanding of transcription and epigenetic basis of kidney injury. Yet, sequencing remains very costly and limit the number of ChIP-seq experiments feasible in a typical research lab. As sequencing costs come down and improvements are made in targeted sequencing, a much larger number of genes and histone marks could be screened simultaneously and over more time points. Although most of the genes we examined are primarily expressed in renal tubules, the cellular heterogeneity of the kidney could have compounded interpretation of the results. In this regard, future application of cell type enrichment, single cell and NGS technologies should facilitate transcription and epigenetic studies in enriched fractions of renal tubule, endothelial and other specific cell types isolated from injured kidneys. Finally, an important limitation of our study was using relatively few models for AKI and CKD. It is possible that other renal injury models may be associated with different transcriptional and epigenetic profiles^88^. Ultimately, integrative approaches using animal models of kidney disease must be scaled up to human-based studies to establish clinical relevance^89^.

## Conclusion

Simultaneous transcriptional and epigenetic profiling across multiple models of acute and chronic kidney injury can yield meaningful insights into the pathophysiology of this complex syndrome. We found that increased transcription was mainly responsible for injury-induced gene upregulation while, post-transcriptional processes appeared to be the more predominant drivers of gene downregulation. There were distinct subsets of genes that shared overlapping epigenetic changes. Epigenetic alterations are pharmacologically reversible and an increasing numbers of small molecules are being introduced that target genome-associated epigenetic factors^81,90^. Further, it is increasingly being recognized that epigenetic drugs can exhibit gene selectivity opening exciting avenues to target sets of specific loci while minimizing off target effects^83^. This study introduces a potential paradigm for testing rationally designed epigenetic interventions.

### Key points of the study

- Transcriptional control plays a key role in the upregulation of both AKI and CKD genes.
- Post-transcriptional control could be playing a more significant role in decreased expression of both AKI and CKD genes.
- Transcriptionally upregulated genes share more epigenetic similarities than downregulated genes.
- Transcription and epigenetic similarities at kidney injury related genes could be exploited to develop therapies that selectively target and correct expression of these genes during AKI and CKD.

## Methods

### Reagents

Bovine serum albumin (BSA), phosphate buffered saline (PBS), salmon sperm DNA, and protein A were from Sigma, and proteinase K was from Invitrogen. Formaldehyde, ethanol, NaCl, EDTA, Triton X-100, NP-40, Tris, leupeptin, PMSF, p-nitrophenyl phosphate, NaF, Na_3_VO_4_, Na_2_MoO_4_ and β-glycerophosphate were from Sigma. The antibodies were commercially available and are listed in Table S3.

### Mouse models

All animal studies were performed in accordance to the NIH Guide for the Care and Use of Laboratory Animals and were approved by the Institutional Animal Care and Use Committee (IACUC) at the University of Washington, Seattle Children’s Research Institute and Fred Hutchinson Cancer Research Center. Male SD-1 and C57BL/6 mice 8–10 weeks of age, 20–35 gm were used.

#### Acute Ischemia-Reperfusion (IR) and Lipopolysaccharide (LPS)

The experimental protocols have previously been described^25,26,64,65,68^. In brief, mice were anesthetized with pentobarbital and subjected to a midline abdominal incision under sterile conditions. Left renal ischemia was induced with an atraumatic microvascular clamp applied to the renal pedicle. After 30 min of unilateral renal artery occlusion, the clamp was released and reperfusion of the entire kidney was assessed visually (by loss of global cyanosis). Twenty four hours after IR injury, mice received a tail vein injection of either LPS (2 mg/kg; 0111:B4; l-2630; Sigma, St. Louis, MO; in 80 μl of saline) or saline. Two hours after injections, mice were re-anesthetized, the abdominal cavity was opened, the kidneys were harvested and rapidly frozen at −80 °C. The mice died within 30 sec due to exsanguinations. Thus, the timing of sacrifice relative to IR was 26 hrs, and 2 hrs post either LPS or saline treatment. As previously documented^27^, the right non-ischemic, contralateral (control) kidney recapitulates what is seen in sham operated kidneys and hence served as an internal control for IR, LPS and IR + LPS injured kidneys. Histology for IR and LPS models of AKI have previously been evaluated^44,64,91^.

#### Mouse model of chronic IR and UUO

Unilateral ureteral obstruction (UUO) and unilateral ischemia reperfusion injury was performed as previously described^29^. Mice were sacrificed 14 days post UUO. In brief, chronic unilateral ischemia reperfusion injury (IR) model was performed by placement of a vascular clamp on the left renal pedicle for 28 minutes while mice were kept at a constant temperature of 37 °C. Mice were sacrificed 24–26 days post IR. Sham UUO and IR surgeries were performed in the same manner as the disease model surgeries but without tying the ureter and clamping the vascular pedicle, respectively. Contralateral, Sham, UUO and IR kidneys were harvested and rapidly frozen (−80 °C). Histology for chronic UUO and IR models have previously been evaluated^29,92^.

### RNA Extraction and cDNA Synthesis

RNA was extracted from tissue fragments using Trizol reagent as per the manufacturer’s protocol. To synthesize cDNA, 400 ng of Trizol-extracted total RNA was reverse transcribed with 200 units MMLV reverse transcriptase (Invitrogen) and oligo dT primers in 10 μl reactions in 96-well microplates. RT reactions were diluted 100-fold prior to running qPCR^93^. RT-qPCR primers are listed in Table. S1.

### Chromatin preparation and multiplex Matrix ChIP platform

The multiplex microplate Matrix ChIP method was previously described^23,24^. Briefly, for ChIP assays, tissue fragments (10–20 mg) were cross-linked with formaldehyde, and chromatin was sheared using Diagenode Bioruptor. ChIP assays were done using protein A-coated 96-well polypropylene microplates as described before^23^. 1 μl of eluted DNA was used in 2 μl real-time PCR reactions (ABI7900HT). All PCR reactions were run in quadruplicates. Final results are expressed as fraction of input DNA^24^. Matrix ChIP qPCR primers to 5′ end of genes used in this study are shown in Table S2 and list of antibodies in Table S3 (Supplement).

### Statistics and visualization

PCRCrunch and GraphGrid were used to acquire, store and analyze large data sets generated by microplate RT-qPCR AND Matrix ChIP-qPCR^25,26^. Pair-wise statistically significant differences are represented by the size of a circle for each comparison made with a small circle representing p < 0.05, a large circle indicating p < 0.01 and no circle implying non-significance. GraphGrid uses a two-tailed Student’s t-test to compute p-values^26^.

### Correspondence analysis

Correspondence analysis, a form of multidimensional scaling similar to principal component analysis, was applied to the entire gene expression dataset to assess whether global variations in transcription distinguished acute from chronic kidney injury^94^. To allow easier visualization, we averaged gene expression values for each condition (n = 6 mice) to display the five experimental conditions utilized (acute: LPS 2 h, I/R 26 h, I/R + LPS 26 h; chronic: I/R 24d, UUO 14d). The first three orthogonal axes are shown in Fig. 1, each of which captures part of the overall transcriptional variability across conditions.

### Heatmap and functional enrichment analysis

RT-qPCR and ChIP-qPCR values during injury conditions were normalized to their uninjured contralateral controls and were log_2_-transformed. Heatmaps were generated using Multiple Experiment Viewer (MeV-TM4) program on log_2_-transformed qPCR and ChIP-qPCR data. For each subset of genes with distinct expression pattern during AKI and CKD, we performed Gene Ontology analysis using Database for Annotation, Visualization and Integrated Discovery (DAVID, v6.8)^95^. Only genes with at least 1.5 fold up or down-regulation in response renal injury (AKI and/or CKD) were selected for this analysis and an FDR ≤ 0.05 was used to designate significant enrichment for a given GO category. Although the 120 candidate genes were pre-selected based on their association with kidney injury and therefore did not represent an unbiased sample, the rationale for the GO analysis was to assess whether distinct functional enrichment profiles emerged based on transcriptional patterns in acute vs. chronic renal injury.

## Electronic supplementary material

Supplement
