# Supplementary material for: Distinct patterns of transcriptional and epigenetic alterations characterize acute and chronic kidney injury
Source: Sci Rep. 2018 Dec 14;8:17870. doi: 10.1038/s41598-018-35943-x (PMC6294783; doi:10.1038/s41598-018-35943-x)
Supplement: Supplementary file 1 — Supplement [file 41598_2018_35943_MOESM1_ESM.pdf]

SREP-17-56516A

Supplementary information

Distinct patterns of transcriptional and epigenetic alterations characterize acute and chronic kidney injury

[1*]Roya Sharifian, [2*]Daryl M. Okamura, [1]Oleg Denisenko, [4]Richard A. Zager, [4]Ali Johnson, [1,3]Sina A. Gharib, and

[1]Karol Bomsztyk,

[1]UW Medicine Lake Union, [3]Computational Medicine Core, Center for Lung Biology, University of Washington, Seattle, WA 98109, USA. [2]Seattle Children's Research Institute, Center for Developmental Biology & Regenerative Medicine, University of Washington, Seattle, WA 98105, USA. [4]the Fred Hutchinson Cancer Research Center Seattle, WA 98109, USA.

*These authors contributed equally to this work

Address for correspondence:

Karol Bomsztyk

UW Medicine at Lake Union

Box 358050

University of Washington,

Seattle, WA 98109 USA

Tel. (206) 616-7949

Fax. (206) 616-8591

e-mail: karolb@u.washington.edu

Table S1. PCR primers RT-qPCR

Primer	Entrez ID	FWD Sequence	REV Sequence	Primer	Entrez ID	FWD Sequence	REV Sequence
Acta2	11475	GACTCTCACACGTCATCACT	AACTGCATTTTTGGGGACAC	Kdm6b	216850	ACTTTCTTGGACCAGTACCC	TTCAGTTCCCACTTCTTCCC
ActB	11461	TCTCAGCTGTGGTGGTGAAG	GTCCCTGTATGCCTCTGGTC	Kdr	16542	CGCTCACCTCCTGTTTAAATG	CACCTCTCTCGTGATTTCCA
Actc1	11464	ACCGCAAGTGCTTCTGAGAT	GTTGCAAGTCCTGGTCTGGT	Kim1	171283	TTGCCTTCCGTGTCTCTAAG	AGATGTTGTCTTCAGCTCGG
Actn4	60595	CTTCTCCACAGCCCTCTATG	CAAGGGAGTCTCTGTTCAGG	Klf2	16598	TGACTTATTTCCCTCGGGTC	ACGTTGGGGACAGTAAACTC
Agt	11606	GCGCTGACCGAGAATAAATG	GTCACCCCAGTATCCAAACA	Klf4	16600	AGGAAGCGATTCAGGTACAG	CCCCGTTTGGTACCTTTAGA
Srp14	20813	GCCTTTAATCCCAGCACTTG	CTCTGTGTAGCCCTGGCTGT	Klotho	16591	TTTGTCATGGGTGGTAGAGG	TCAGATACCATTCACCCTGC
Angpt1	11600	AGTTGGAACAGCCCATTGTA	TGAAGGCCTACGAACACTTT	Kmt1b	64707	ACTTCTGGGGATTAGATGCG	TTAGAACTCCCAGACCCAGA
Angpt2	11601	AAGAATGTTCCGTGGAGTT	TGCTTAGAGGAATGTGGTCC	Kmt1c	110147	CATCAACACCTGAGGACTCT	TTACTGGGGTCTCTGATGGA
Aoc1	76507	CAACGGTCTCAACCATGTTC	GGCTTGTAGGTCCCATTGTA	L1l2	14573	CCCAAAACATCCAGGAAATC	TGTTCATGGGCATCTCTTTCT
Apoe	11816	GAACAGACCCAGCAAATACG	TCTTCCACTATTGGCTCGAA	L32	19951	TTAAGCGAAACTGGCGGAAAC	TTGTTGCTCCCATAACCGATG
Arg1	11846	TACCAGAAAGGAACTGCTGG	CACATGAAAACCATCTGGGG	Lama1	16772	AGTCCTTGGACCTGAGCCGA	GCTGGCGACAGTTTAGGGCT
Axin2	12006	GGCCTGTGTATAGTGTAGGG	CAGGTACTGTCCTGATTCCC	Lama2	16773	CTGAGGGGTGTTCAACCTGT	CTGACACCGTGCACAAAGAA
Bax	12028	CATGGCAGACAGTGACCATC	GAAAAATGCCTTTCCCCTTC	Lama3	16774	AGAAGTCACCTTTGGGAGCA	GATTGGGGGAAAAAGCTCTC
Bcl2l1	12048	GCTGGTGCCCAGAGACTGAC	GCCCCTCAGAAGCCAGAACC	Lama5	16776	GAGGATGCTTGAGGAAGCTG	AAGGTCTGGGAACCCTCAAT
Bmp7	12162	GTCTCAGGAAGAGCTAGTGG	TCGACGACAGCTCTAATGTC	Lamb1	16777	AGAATTGGTGCGACTGGAAG	TAGATGACTGCCTCGGTTTG
Clu	12759	ACCCCTAGAGAACTCCACAT	TGCAGGCATTAGTGTACAGA	Lamb2	16779	GGAGGGCACATATGAGGAGA	GACGATCGTGTGCAGAACAG
Col11a1	12814	ACTGCTTTAACATCCACCCA	TGGCATTACTGAAGCACTCT	Lamb3	16780	GGGCGAGTGCTCTACTATGC	ACAGGCTCAGTGGGAACAGT
Col13a1	12817	ATGCCTCTAACCTGGGACT	TGGGCATTTTCAAAAGGTGAA	Lamc1	226519	GGAACCTTCCCACACGGGTC	CGGTGCTGATGCCCTCAAGT
Cox2	19225	TGCCTCCCACTCCAGACTAGA	CAGCTCAGTTGAACGCCTTTT	Lamc2	16782	TGGCAGTGTGGATGAAGAAG	GGCAGGTTGTCTCGAATGTT
Cst3	13010	AAGGGCTGAGTCTAGAAGGA	CCTTCTGCGAGATGAAACAC	Lamc3	23928	AGATCAAGCAGTGCCCAGAT	AGATCAAGCAGTGCCCAGAT
Ctgf	14219	TGCTGTGCAGGTGATAAAGC	CCACCCCAAACCAGTCATAA	Ldlr	16835	GTCCTGCTGTGGAGGAACTG	CAGGCTGACCATCTGTCTTG
Ctnnb1	12387	GATGGACCCTATGATGGAGC	CAGCCCATCAACTGGATAGT	Lrp5	16973	CTACCTGGACTTGAATTCGGA	TGCAGATAGGTACTGGCTGT
Cubn	65969	TGCTTCATCAAATCGGGTCT	CCGATTCAGCTACTCCACAT	L-FABP	14080	CAAGCTGGAAGGTGACAATAA	GTGTCTCCATTGAGTTCAGTC
Cxcl1	14825	TGCATCTGGATAGGGAAAGG	ATCAGGCAATGAACCAGAGG	Mbd3	17192	TGGACCTTGAACTCACTTGT	TCACTGCTCATCTGCTTTCA
Cypa	268373	GTGGTCTTTGGGAAGGTGAA	TTACAGGACATTGCGAGCAG	Mcp1	20296	TTGAATGTGAAGTTGACCCG	TTAAGGCATCACAGTCCGAG
Dvl1	13542	CACACAGTACCAAGTGGGTC	CCTGACTTCGAGGGCTACTG	Mkp1	19252	CAGCTGCTGCAGTTTGAGTC	GGTGATGGGGCTTTTAAGGT
Edn1	13614	GCATCAAGGCATCTTTTCGT	GTAGTCAATGTGCTCGGTTG	Muc1	17829	CAACTACGTAAGCCTGGTGA	CTGAGAGCCACCACTAACTG
Egf	13645	ACTGGGAGTAGACAGAAGGT	AGTGATTAGCCGTGGAAACT	Myc	17869	ACATCCTGTCCATTCAAGCA	AACTGTTCTCGTCGTTTCCT
Egfr	13649	CTGCCGTGTCAAAGAAGAC	TGCGTCATCTATGTTGTCCT	Neph1	170643	CCTATGACCTTCGCTGGCCT	AGGCTCCCCAGCATCCATGT
Egr1	13653	AACAGCCCTTTCACTTACCA	CTTGGACATGGCTGTTTCAG	Netrin1	18208	CCCTTTGCTTACCATTTGGG	CACATACCTTTGTGCCACTG
Epo	13856	AATTGATGTCGCCTCCAGAT	AGAGCTTGCAGAAAGTATCCA	Ngal	16819	AGATGCTCCTTGGTATGGTG	CTGTCTGCCACTCCATCTTT
Ezh2	14056	TGAAGTATGTGGGCATCGAA	GAAGCTAAGGCAGCTGTTTC	Nos3	18127	ACCCAAACCTTGTGAGTTAC	CTGGGACTCACTGTCAAAGA
Fasn	14104	CCCTGGTGTCCTCCTCAGT	CGACGCCCTCATCTCTGT	Nphs2	170484	GCTTAGGTGCTCCTGGATGA	GACTCAGTCTTGCCCCTCTG
Fgf10	14165	GCCACCAACTGCTCTTCTTC	CTGACCTTGCCGTTCTTCTC	Oct4	18999	CACCACACTCTACTCAGTCC	CTGGTGCCTCAGTTTGAATG
Flt1	14254	GACATGGGAAGACAGGGTAG	AGCCATTTTAGAGACCCAGG	p21	12575	GAAAACGGAGGCAGACCA	TGACCCACAGCAGAAGAGG
Fn1	14268	GCCAACGCTTTACTACTGTG	TGACGTCCACATCATGGAAT	p53	22059	CTCTGAGTAGTGGTTCCTGG	TGGCTGGATAGAATTTCGCT
Foxo1	56458	GTCTCCCGGTACTTCTCTGCT	GTGGTCGAGTTGGACTGGTT	Pai1	18787	TGAGAGAGGGCAAAGTGGTT	ATACAGCAGCCGGAAATGAC
Fsp1	20198	GGAGGCCCTGGATGTAATTGT	TGTCACCCTCTTTGCCTGAGT	Pdgfb	18591	CCTCTGATCTTTTGCGGTTC	CTCCCCCACTTCCAATCTAC
				Pdgfrb	18596	TTTTCATTCAGAGCCCACCT	ATTCATTAAGCATGGCTGGC

Fyn	14360	GGGGGTTGTACACGTCCCAT	CTGGAGCACTCCTGACACGG	Pecam1	18613	TGCCTTGTTCATGTTGGGTA	TCTCCTGGAACCTCCTTTCA
Fzd1	14362	GAGAGAGACAAACACGACCT	ACAAGATGGGATGCAGTAGG	Pnmt	18948	ATCTTACTAGCTTCCAGCGG	CAATGAGGAGGAGATGACCC
Gapdh	14433	AACTTTGGCATTGTGGAAGG	AGTGGATGCAGGGATGATGT	Rage	11596	GATGCAAAGGCAATCTCACTCCTGCAT C	CCTGGTATGGTGGGAGGCATAG
Grp	225642	GTGGCAGAGCTCCTGAGAAC	ACCAGAGCCAACAGCAAGAG	Sema3a	20346	GCGGTGGCTTATGTACTACT	GGAACAATTTACGACCTGGC
Gsk3a	606496	CCCCCTGGGTGTAAATAGAT	GAGGACAAGAAGGGGTACAG	Sox2	20674	TTCTAGTGGTACGTTAGGCG	CCCGGAGTCTAGCTCTAAAT
Hamp	84506	GGATGTGGCTCTAGGCTATG	CTTCCCCATCTGCATCTTCT	Spp1	20750	CATGAAGAGCGGTGAGTCTA	TTGTTGTCCTGATCAGAGGG
Hdac9	79221	CCCTGCCCAATATCACTCTG	CCCTGCCCAATATCACTCTG	Tek	21687	AGGAAGAAAAGCGAGGGAAA	CCCTCTCTTCTGCTACTTGG
Hif1a	15251	CTGCGTGCATGTCTAATCTG	CGCGGAAAACTCTTGTTTCA	Tgfb1	21803	GCAGTGGCTGAACCAAGGA	GCAGTGAGCGCTGAATCGA
Hmgcr	15357	TGTTCAAGGAGCATGCAAAG	CTTACCTGTTGTGAACCATGTG	Timp1	21857	TGCCAGACTAACTGCTCACG	TTAAAGGCTTGTGGGTTTGG
Hmgcs1	208715	CAAGGTGAACTTCTCTCCACC	ACACCGGGCATACTTCCTG	Timp2	21858	AGAAACGGTTAAGGACTCCC	TGTCCTCCCAGTCTGTCTTA
Hmox	15368	TCTTGCCTGGCTCTCTTCTC	GGCTGCTGGTTTCAAAGTTC	Timp3	21859	CAATCAGTCAAAGGCAGCAA	TCCCTCTGACATGCACACAT
Hpx	15458	GGGCCCAATTTGTACTGCTA	CCAAGGATGCTGTTCACCTT	Tirap	117149	ATATCCCCAGGGAGAGACAA	TCAAGGTACATACCAGGGGA
Icam1	15894	CGAGGGTTTCTCTACTGGTC	TGCCAGTCCACATAGTGTTT	Tlr1	21897	GGCAAACCTAAGAGCATCCA	ATTGCTGTGTGTAACACGTTC
Ifng	15978	GCTTTAACAGCAGGCCAGAC	GGAAGCACCAGGTGTCAAGT	Tlr2	24088	TGCTTTCCTGCTGGAGATTT	TGTAACGCAACAGCTTCAGG
Igf1	16000	TGCCTCTAAAGAGCCGAAAA	GAGCTACGTGGGAAGAGGTG	Tlr4	21898	GCATGGCTTACACCACCTCT	GTCTCCACAGCCACCAGATT
Igfbp7	29817	AATCCATGAGCCTCTGTAGC	AAGAGAAGTGTGTCAGGCAA	Tnf	21926	CGCTACATCACTGAACCTCT	TTCTCTCAATGACCCGTAGG
Il10	16153	CCAGGGAGATCCTTTGATGA	AACTGGCCACAGTTTTCAGG	Vcam1	22329	TGCAAGGAGCTAACCAGAAA	ATCATGGGACCATTCCAGTC
Il18	16173	GTGTTCCAGGACACAACAAG	CTTCCTTTTGGCAAGCAAGA	Vegfa	22339	GAGAGAGGCCGAAGTCCTTT	TTGGAACCGGCATCTTTATC
Il6	16193	GTGGCTAAGGACCAAGACCA	ACCACAGTGAGGAATGTCCA	Vim	22352	CGCCATCAACACTGAGTTCAA	TGGCAAAGCGGTCATTCA
Junb	16477	GCCTTTCTATCACGACGACT	AAGGTGGGTTTCAGGAGTTT	Wnt1	22408	TGTTGCGGTTCCTGATGTAT	CTATAGGGACCCGAGAGACA
Kdm6a	22289	AGTTCTGCACCACTGTATT	TTGACACTCCACACTTGGAA	Ym1	12655	AGCATGGTGGTTTTACAGGA	CTGTACCAGCTGGGAAGAAA

Table S2. PCR primers Matrix ChIP-qPCR

Primer	Entrez ID	FWD Sequence	REV Sequence
Apoe Exon1	11816	CAATTTTTCCCTCCGCAGAC	GTCAGCAATGTGACCAACAG
Epo Exon1	13856	GTGATCTGGCCCTACAGAAC	GAGCTCCTTAGCGACCC
Hmox Exon1	15368	GAGCCTGAATCGAGCAGAAC	AGCTACAGGTAGACTGGGCG
Hpx Promoter-Exon1	15458	GGGTGGTGCTGTTACCAGTT	CCCAGCAACACCAGGATATT
Igfbp7 Exon1	29817	GCATCCAGCCACCTTATGAT	AAGAGGAGGAAGAGGAGAGG
Kdr Exon1	16542	GAGTCTGTGCCTGAGAACTG	CAGAACCACAGAGCGACA
Kim1(Havcr1) Exon3	171283	TGGAGATTCCTGGATGGTTT	TAGAAGCTTACCTGGTTTAACTTG
Klotho Exon1	16591	CGCCTACCAGACCGAGG	GTGGGTGAACGTATCCCAG
Kmt1b (Suv39h2) Exon1	65707	CAGCGAGCGTGGTCC	CTTGAATAAGGCACCTCGTG
Lrp5 Exon2	16973	CTTCCAGTTCTCCAAGGGTG	TCTGGTTCAGGTAGGTCTGT
Ngal (Lcn2) Exon1	16819	ACTCAGAACTTGATCCCTGC	CCTTCAGGGTCCTACCTGAT
Pnmt Exon1	18948	GCCTATCTCCGCAACAACTA	CAAAGACTTGTGCCATGCAG
Spp1 (Osteopontin) Intron1	20750	GGCTGAATTCTGAGGGTGAG	AAAGACCAGAACAGCACGAG
Timp1 Exon1	21857	TGCCAGACTAACTGCTCACG	TTAAAGGCTTGTGGGTTTGG
Timp2 Exon1	21858	GATCCTCGGAGCGCAATAA	CTCCTCCGCTGCCTTCTATG
Tlr4 Exon1	21898	GAAAATGCCAGGATGATGCC	ATTCAAGCTTCCTGGTGTCA
Tnf Exon1	21926	AGTGCCTCTTCTGCCAGTTC	GCAGGTTCTGTCCCTTTCAC
Vcam1 Exon1	22329	GGTCAGGAAAAGCCAGAGAT	GGGCTGAAGTCATTCTGAGT

Table S3

**Antibody**	**Catalogue No**	**Source**	**Manufacturer**
Pol II CTD (4H8)	sc-47701	Mouse monoclonal	Santa Cruz
H3K9me2	720092	Rabbit polyclonal	ThermoPierce
H3K9me3	701784	Rabbit monoclonal	ThermoPierce
H3K14me2	MA3-059	Mouse monoclonal	ThermoPierce
H3K27ac	720096	Rabbit polyclonal	ThermoPierce
H3K4m1	OAAH00066	Rabbit	Aviva
H3K4m2	OAAH00067	Rabbit	Aviva
H3K4m3	OAAH00068	Rabbit	Aviva
H3K27me3	720069	Rabbit polyclonal	ThermoPierce

Table S4. Gene function	
**Gene**	**Function**
Acta2	alpha-2 actin proteins in smooth muscle; important for contraction
Actb	beta-actin; important for motility and cell shape
Actc1	Actin; defects linked with idiopathic dilated and familial hypertrophic cardiomyopathy
Actn4	Alpha actin found in non-muscle cells; involved in binding actin to the membrane
Ager	Advanced glycosylation end product-specific receptor; Believed to be pro-inflammatory
Agt	Angiotensinogen; regulates balance of fluid and salts in the body and blood pressure
Angpt1	Angiopoietin; involved in angiogenesis
Angpt2	Endothelial cell-derived regulator of angiogenesis and ligand for endothelial-specific receptor tyrosine kinase
Aoc1	Amine oxidase, copper-containing 1; part of the oxidoreductase family
Apoe	Apoliprotein E which binds to a specific liver receptor; essential for catabolism of triglyceride-rich lipoproteins.
Arg1	Arginase; Involved in the urea cycle which processes excess nitrogen to be excreted by the kidneys.
Axin2	Downregulates beta-catenin and is an inhibitor of the Wnt signaling pathway.
Bax	Apoptosis regulator; Part of Bcl2-gene family.
Bcl2	Mitochondrial-mediated apoptosis inhibitor; Abnormal expression has been associated with cancer.
Bcl2l1	Regulates programmed mitochondrial-mediated apoptosis during development and tissue homeostasis.
Bmp7	Bone morphogenetic protein 7; ligand of TGF-beta used in gene regulation. Abnormal expression associated with developmental defects including kidney defect.
Ccl2	Chemokine (C-C motif) ligand 2; chemotactic activity for monocytes and memory T cells
Cdkn1a	Cyclin-dependent kinase inhibitor 1A; Cell cycle regulator at the G1 phase.
Chil3	Chitinase-like 3; Possible involvement in inflammation and allergy.
Clu	Clusterin; Possible involvement in cell death, tumor progression and neurodegenerative disorders.
Col11a1	Alpha-1 subunit of type XI collagen; Necessary for embryonic cartilage development.
Col13a1	Alpha-1 subunit of type XIII collagen
Cst3	Cystatin-C; Cysteine protease inhibitor; Inhibits beta-amyloid aggregation; Possible biomarker for atherosclerosis
Ctgf	Connective tissue growth factor; Involved in many biological processes including cell adhesion, migration and angiogenesis.
Ctnnb1	Beta-catenin; Involved in cell adhesion, cell communication and the Wnt signaling pathway.
Cubn	Cubilin; Primarily found in the kidneys; involved in reuptake of proteins and vitamins (e.g. vitamin B12) that would otherwise be lost in urine.
Cxcl1	Chemokine ligand 1; Possible association with neutrophil-recruitment; Deficiency in mouse was linked to colitis.
Dusp1	Dual specificity phosphatase 1; anti-inflammatory functions
Dvl1	Dishevelled segment polarity protein 1; Regulates Wnt signaling and planar cell polarity pathways.
Edn1	Endothelin 1; primarily involved in vasoconstriction; therapeutic target for pulmonary arterial hypertension.
Egf	Epidermal growth factor; Involved in cell proliferation and differentiation.
Egfr	Epidermal growth factor receptor
Egr1	Early growth response protein 1; transcription regulator; possible tumor suppressor

Ehmt2	Euchromatic histone-lysine N-methyltransferase 2; catalyzes demethylation of H3K9
Epo	Erythropoietin; Regulates production of red blood cells; primarily expressed in the liver during fetal development and in the kidney as adults.
Ezh2	Enhancer of zeste homolog 2; transcriptional repressor of tumor suppressor genes by histone methylation.
Fabp1	Fatty acid-binding protein 1; primarily expressed in the liver; involved in binding, transport and metabolism of long-chain fatty acids. Abnormal expression has been linked to obesity.
Fasn	Fatty acid synthase
Fgf10	Fibroblast growth factor 10
Flt1	FMS-like tyrosine kinase 1; vascular endothelial growth factor receptor family; involved in angiogenesis and vasculogenesis
Fn1	Fibronectin 1; Involved in blood clotting, wound healing, formation of the extracellular matrix, cell migration and cell differentiation.
Foxo1	Forkhead box O1; Involved in the regulation of gluconeogenesis and glycogenolysis.
Fyn	Proto-oncogene tyrosine-protein kinase Fyn; Regulates cell growth; Often associated with T cell and neuronal signaling.
Fzd1	Frizzled class receptor 1; Receptors for Wnt signaling proteins.
Gapdh	Glyceraldehyde-3-phosphate dehydrogenase; Many functions including mRNA stability regulation.
Gdnf	Glial cell line derived neurotrophic factor; promotes survival of many types of neurons; involved in kidney development.
Grp	Gastrin releasing peptide; Affects neuroendocrine regulation, gastrointestinal secretion, nociception, cell proliferation and inflammation.
Gsk3a	Glycogen synthase kinase 3 alpha; Regulates glycogen synthase; Involved in the Wnt signaling pathway.
Hamp	Hepcidin antimicrobial peptide; hormonal regulator of iron metabolism.
Havcr1/Kim-1	Hepatitis A virus cellular receptor 1; membrane receptor for hepatitis A virus and TIMD4 (T-cell membrane protein 4).
Hdac9	Histone deacetylase 9; Transcriptional regulator
Hif1a	Hypoxia inducible factor 1; Regulator of cellular and developmental response to hypoxia.
Hmgcr	HMG-CoA reductase; rate-controlling enzyme of the mevalonate pathway which produces cholesterol.
Hmgcs1	HMG-CoA synthase 1
Hmox1	Heme oxygenase 1; essential enzyme in heme catabolism
Hpx	Hemopexin; Deficiency is associated with several inflammatory diseases such as septic shock.
Icam1	Intercellular adhesion molecule 1; participates in innate immune response; binds leukocyte adhesion protein LFA-1
Ifng	Interferon gamma; active in both innate and adaptive immune systems in response to viral and microbial infections.
Igf1	Insulin-like growth factor; promotes development during fetal and postnatal life; predominantly expressed in the liver.
Igfbp7	Insulin-like growth factor binding protein 7
IL10	Interleukin 10; anti-inflammatory cytokine essential for suppressing immune and autoimmune responses.
IL18	Interleukin 18; pro-inflammatory cytokine
IL6	Interleukin 6; inducer of the acute phase in an inflammatory response; plays role in maturation of B cells.
Junb	Jun B proto-oncogene; transcription factor involved in gene regulation after the primary growth factor response.
Kdm6a	Lysine-specific demethylase 6A; primarily linked to demethylation of lysine residues on H3K27.
Kdm6b	KDM1 lysine-specific demethylase 6B
Kdr	Kinase insert domain receptor; encodes one of two receptors of the vascular endothelial growth factor which is responsible for endothelial proliferation, survival and migration.
Kirrel	Kin of IRRE-like protein 1; Expressed in kidney podocytes which are involved in the ultrafiltration of blood.

Kl	Klotho; primarily found in the kidney; involved in phosphate homeostasis, bone development and bone strength maintenance.
Klf2	Kruppel-like factor 2; primarily expressed in adult lungs; Involved in lung development, embryonic erythropoiesis and adipogenesis.
Klf4	Kruppel-like factor 4; primarily expressed in the gut; Involved in cell proliferation, differentiation and apoptosis.
Lama1	Laminin subunit alpha 1; Involved in cell adhesion, differentiation, migration, signaling, neurite outgrowth and metastasis.
Lama2	Laminin subunit alpha 2
Lama3	Laminin subunit alpha 3
Lama5	Laminin subunit alpha 5
Lamb1	Laminin subunit beta 1
Lamb2	Laminin subunit beta 2
Lamb3	Laminin subunit beta 3
Lamc1	Laminin subunit gamma 1
Lamc2	Laminin subunit gamma 2
Lamc3	Laminin subunit gamma 3
Lcn2/Ngal	Lipocalin-2; biomarker of kidney injury
Ldlr	Low density lipoprotein (LDL) receptor; LDLs are the primary carriers of cholesterol in blood.
Lrp5	Low density lipoprotein receptor-related protein 5; Involved in Wnt signaling pathway which is important for cell proliferation, adhesion and migration.
Mbd3	Methyl-CpG binding domain protein 3; transcriptional repressor
Muc1	Mucin 1; Component of mucus; Involved in cell signaling and kidney development.
Myc	Myelocytomatosis oncogene; Plays role in cell cycle progression, apoptosis and cellular transformation.
Nek1	NIMA (never in mitosis gene a)-related kinase 1; Possible involvement in meiosis, polycystic kidney disease and amytrophic lateral sclerosis.
Nos3	Nitric oxide synthase 3; Regulates vascular tone, cellular proliferation, leukocyte adhesion and platelet aggregation in the cardiovascular system.
Nphs2	Podocin; Assists with filtration in the kidneys and podocyte cell signaling.
Ntn1	Netrin 1; Thought to be involved with developmental axon guidance and cell migration.
Pdgfb	Platelet derived growth factor B; Involved in cell growth, proliferation, differentiation and movement.
Pdgfrb	Platelet derived growth factor receptor B
Pecam1	Platelet/endothelial cell adhesion molecule 1; plays role in removing aged neutrophils from the body, leukocyte migration, angiogenesis and integrin activation.
Pnmt	Phenylethanolamine-N-methyltransferase; primarily expressed in the adrenal medulla; converts norepinephrine to epinephrine.
Pou5f1	POU domain, class 5, transcription factor; regulates cell specific differentiation pathways; plays key role in embryonic development and stem cell pluripotency.
Ppia	Peptidylprolyl isomerase A; Involved in many biological processes including apoptosis and inflammation.
Ptgs2	Prostaglandin-endoperoxide synthase 2; upregulated during inflammation
Rpl32	60 S Ribosomal protein L32
S100a4	S100 calcium binding protein A4; Involved in many cellular processes including cell cycle progression and differentiation.
Sema3a	Semaphorin-3A; important for normal neuronal pattern development; can function as a chemorepulsive agent, by inhibiting axonal growth, or as a chemoattractive agent, stimulating apical dendrite growth.
Serpine1/PAI-1	Serine peptidase inhibitor; fibrinolysis inhibitor; high protein concentrations can lead to thrombophilia
Sox2	Sex determining region Y-box 2; Necessary to maintain pluripotency of undifferentiated embryonic stem cells.

Spp1/osteopontin	Secreted phosphoprotein 1; Osteopontin; Many functions including bone remodeling, biomineralization, increased myocyte apoptosis, cell attachment, wound healing and chemotaxis.
Srp14	Subunit of a ribonucleoprotein; mediates secretory proteins movement to the endoplasmic reticulum
Suv39h2/Kmt1b	Suppressor of variegation 3-9 homolog 2; Histone-lysine N-methyltransferase; exclusively found in testis among normal tissue but is upregulated in many cancers including lung, cervical and bladder cancer. http://www.sciencedirect.com/science/article/pii/S1936523315000595
Tek/Tie2	Endothelial-specific receptor tyrosine kinase; Important for angiogenesis.
Tgfb1	Transforming growth factor beta 1; Regulates cell proliferation, differentiation and growth; Plays role in regulation of other growth factors including interferon gamma and tumor necrosis factor alpha.
Timp1	Tissue inhibitor of metalloproteinase 1; Inhibits extracellular matrix degradation; Promotes cell proliferation in many cell types; Possible anti-apoptotic functions.
Timp2	Tissue inhibitor of metalloproteinase 2; Plays unique role in the Timp family of directly suppressing endothelial cell proliferation; Important for tissue homeostasis maintenance.
Timp3	Tissue inhibitor of metalloproteinase 3; Localized to extracellular matrix; Responds to mitogenic stimulation.
Tirap	Toll-interleukin 1 receptor domain-containing adaptor protein; Involved in the TLR4 signaling pathway of the innate immune system.
Tlr1	Toll-like receptor 1; Recognizes diacylated and triacylated lipopeptides in the TLR2 signaling pathway of the innate immune response.
Tlr2	Toll-like receptor 2; Forms heterodimers with other TLRs to recognize pathogen-associated molecular patterns; Possible role in promoting apoptosis in response to bacterial lipoproteins.
Tlr4	Toll-like receptor 4; Mediates innate immune response to bacterial lipopolysaccharide, a component of Gram-negative bacteria.
Tnf	Tumor necrosis factor; Proinflamatory ccytokine that plays a role in the innate immune response and regulating homeostasis; Possible role in diseases of chronic inflammation.
Trp53/p53	Transformation related protein 53; tumor suppresor
Vcam1	Vascular cell adhesion molecule 1; Involved in adhesion of lymphocytes, monocytes, eosinophils and basophils to vascular endothelium.
Vegfa	Vascular endothelial growth factor A; Induces vascular endothelial cell proliferation and migration; Essential for angiogenesis; Upregulated in many known tumors and expression correlates with tumor stages.
Vim	Vimentin; Component of the cytoskeleton; Organizer of several proteins involved in attachment, migration and cell signaling.
Wnt1	Wingless-type MMTV integration site family, member 1; Plays a role in several developmental processes such as cell fate regulation during embryogenesis; Possible involvement in CNS development

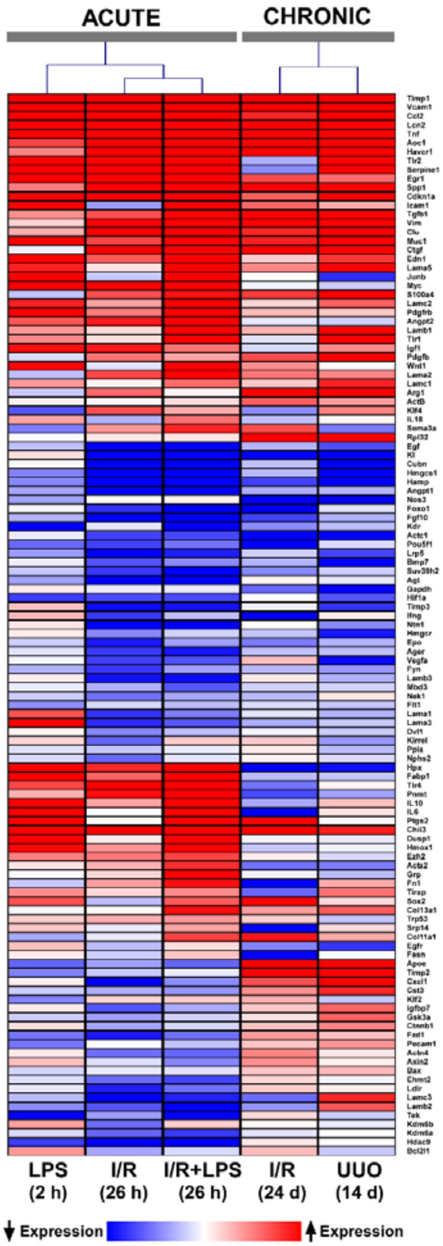

Fig. S1. Integrated analysis of mRNAs encoding renal injury pathways and biomarkers from AKI and CKD models.

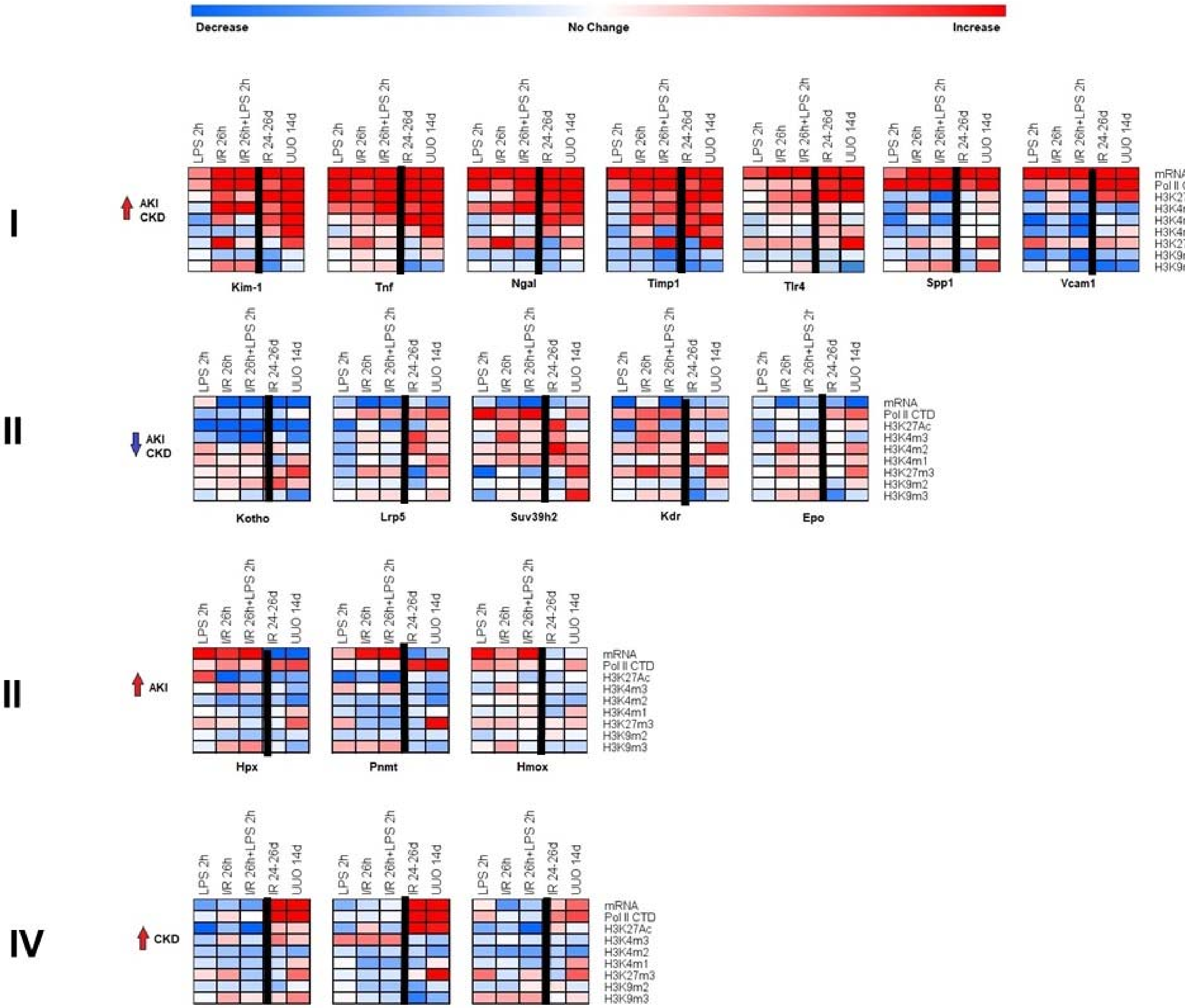

Decrease
No Change
Increase
I
AKI
CKD
Kim-1
Tnf
Ngal
Timp1
Tlr4
Spp1
Vcam1
II
AKI
CKD
Kotho
Lrp5
Suv39h2
Kdr
Epo
II
AKI
Hpx
Pnmt
Hmox
IV
CKD
LPS 2h
I/R 26h
I/R 26h+LPS 2h
IR 24-26d
UUO 14d
mRNA
Pol II CTD
H3K27Ac
H3K4m3
H3K4m2
H3K4m1
H3K27m3
H3K9m2
H3K9m3

Fig.S2. Integrated transcriptional and epigenetic analysis of selected sets of renal injury genes from AKI and CKD models. mRNA**;** total RNA extracted from the mouse kidneys was used in reverse transcription (RT) reactions and cDNA was used in real-time PCR (qPCR) with gene specific primers (Table S1). mRNA levels of a given gene in each sample was normalized to Rpl32. Matrix ChIP-qPCR; sheared cross-linked chromatin was (prepared from the same kidneys used in RT-qPCR) was analyzed in Matrix ChIP-qPCR using antibodies to RNA polymerase II (Pol II) and antibodies to permissive (H3K27Ac, H3K4m3, H3K4m2, H3K4m1) and repressive (H3K9m2, H3K27m3, H3K9m3) marks. qPCR primers were design to the 5'-end of the genes (Table S2). ChIP signals were calculated as fraction of input. Data represents log2 transformed rations of means from injured kidney over control (n=6 mice for each injury model). Shown are genes whose expression was upregulated in both AKI and CKD (I), downregulated in both AKI and CKD (II), upregulated in AKI but not in CKD III) and upregulated in CKD but not in AKI (IV).

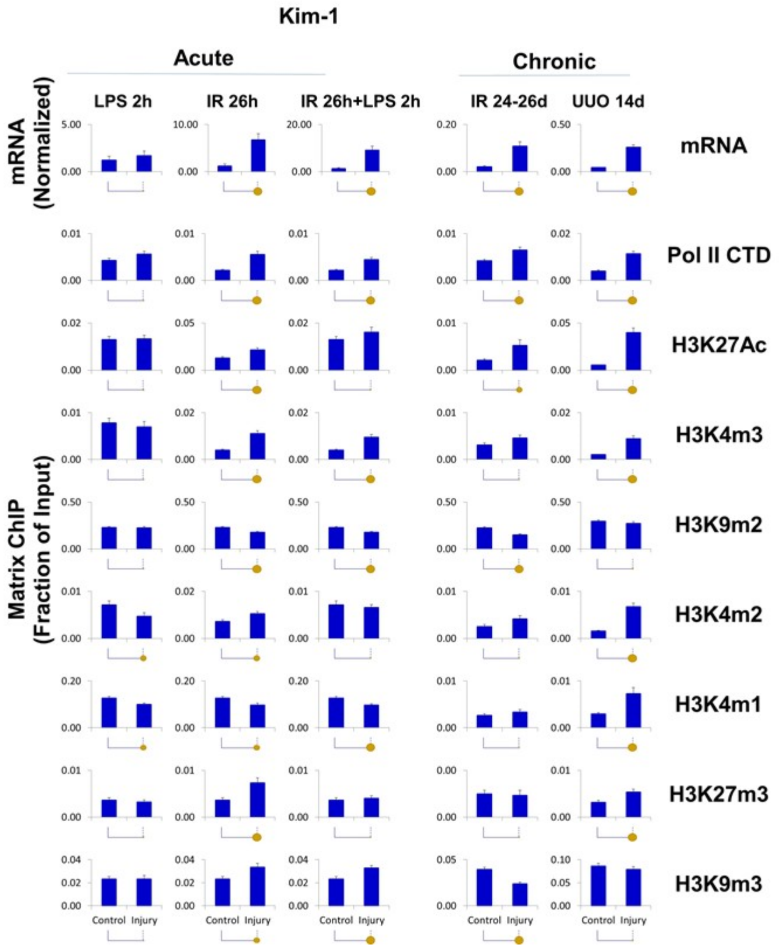

Fig.S3. Transcriptional and epigenetic analysis of Kim-1 (Havcr1) gene in AKI and CKD models. mRNA**;** total RNA extracted from injured (Injured) and control (Control) mouse kidneys was used in reverse transcription (RT) reactions. cDNA was used in real-time PCR (qPCR) with gene specific primers (Table S1). mRNA levels of a given gene in each sample was normalized to Rpl32. Matrix ChIP-qPCR; sheared cross-linked chromatin was (prepared from the same kidneys used in RT-qPCR) was analyzed in Matrix ChIP-qPCR using antibodies to RNA polymerase II (Pol II) and antibodies to permissive (H3K27Ac, H3K4m3, HK4m2, H3K4m1) and repressive (H3K9m2, H3K27m3, H3K9m3) marks. ChIP signals were calculated as fraction of input. Data represents mean , n=6 mice from each group. Statistical differences between two means (p value) are shown by the size of the solid circle under the x-axis: $p<0.05$ by small circle, $p<0.01$ by large circle, and no circle indicating the differences are not statistically significant.

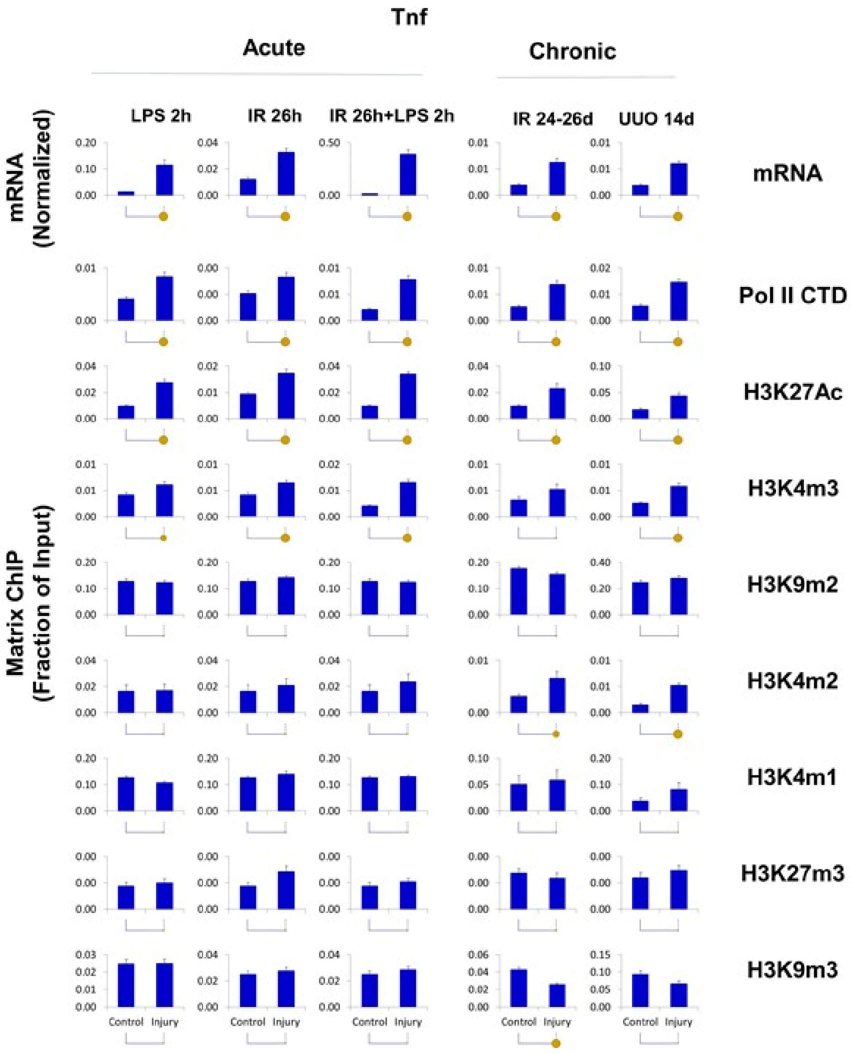

Fig.S4. Transcriptional and epigenetic analysis of Tnf gene in AKI and CKD models.

Transcript and ChIP assays and analysis were carried out as in Fig.S2 using primers to Tnf (Tables S1-2). Data represents mean , n=6 mice from each group.

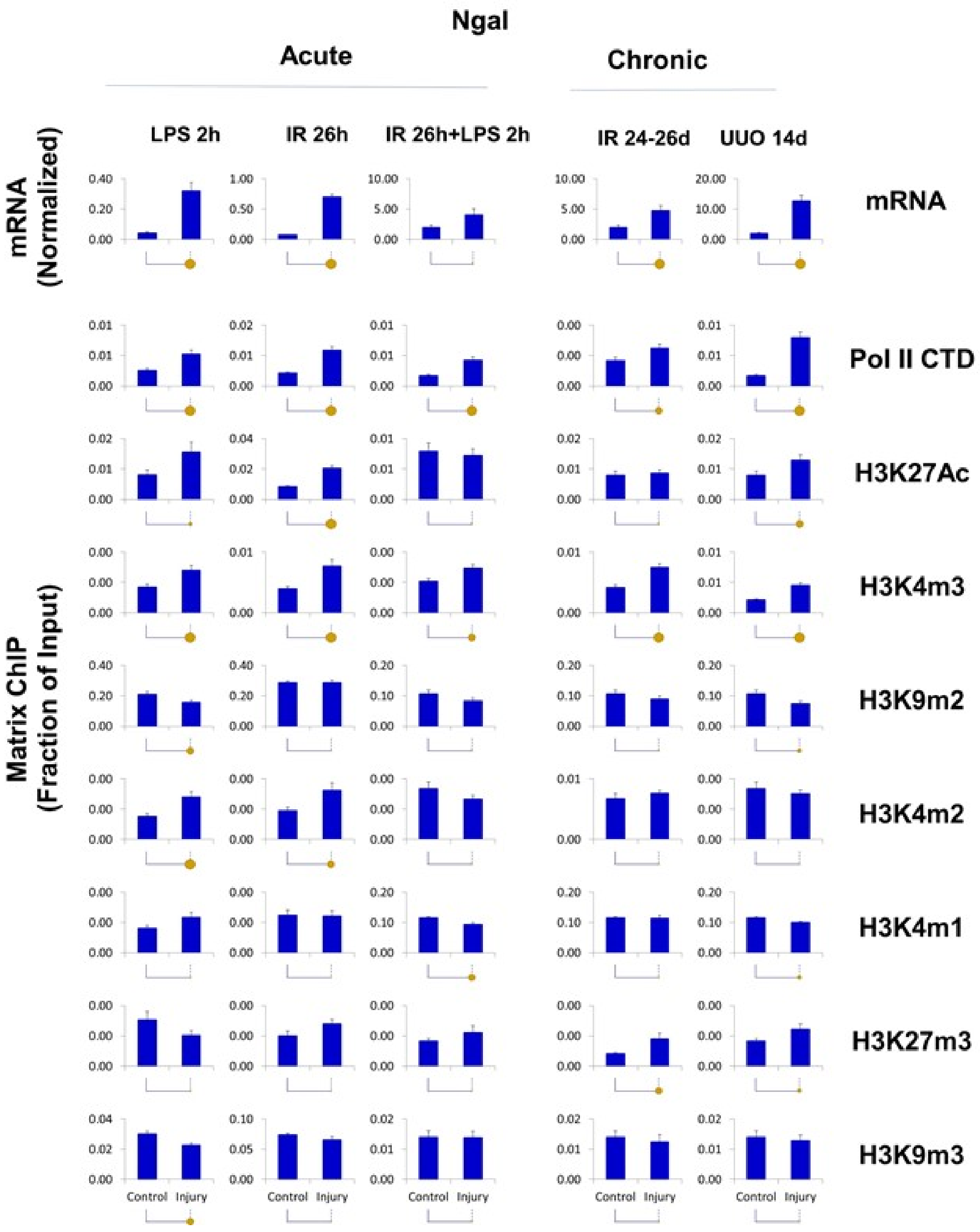

Fig.S5. Transcriptional and epigenetic analysis of Ngal gene in AKI and CKD models. Transcript and ChIP assays and analysis were carried out as in Fig.S2 using primers to Ngal (Tables S1-2). Data represents mean , n=6 mice from each group.

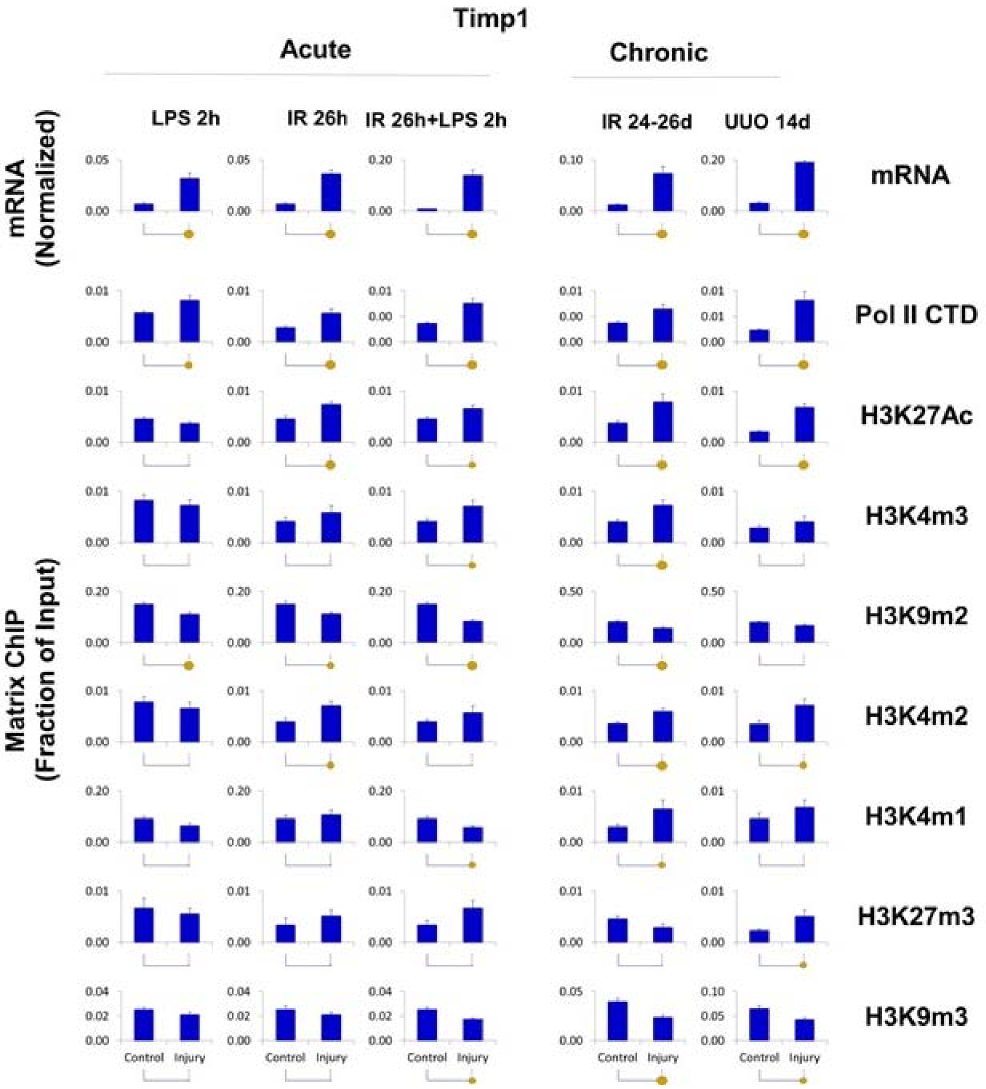

Fig.S6. Transcriptional and epigenetic analysis of Timp1 gene in AKI and CKD models. Transcript and ChIP assays and analysis were carried out as in Fig.S2 using primers to Timp1 (Tables S1-2). Data represents mean , n=6 mice from each group.

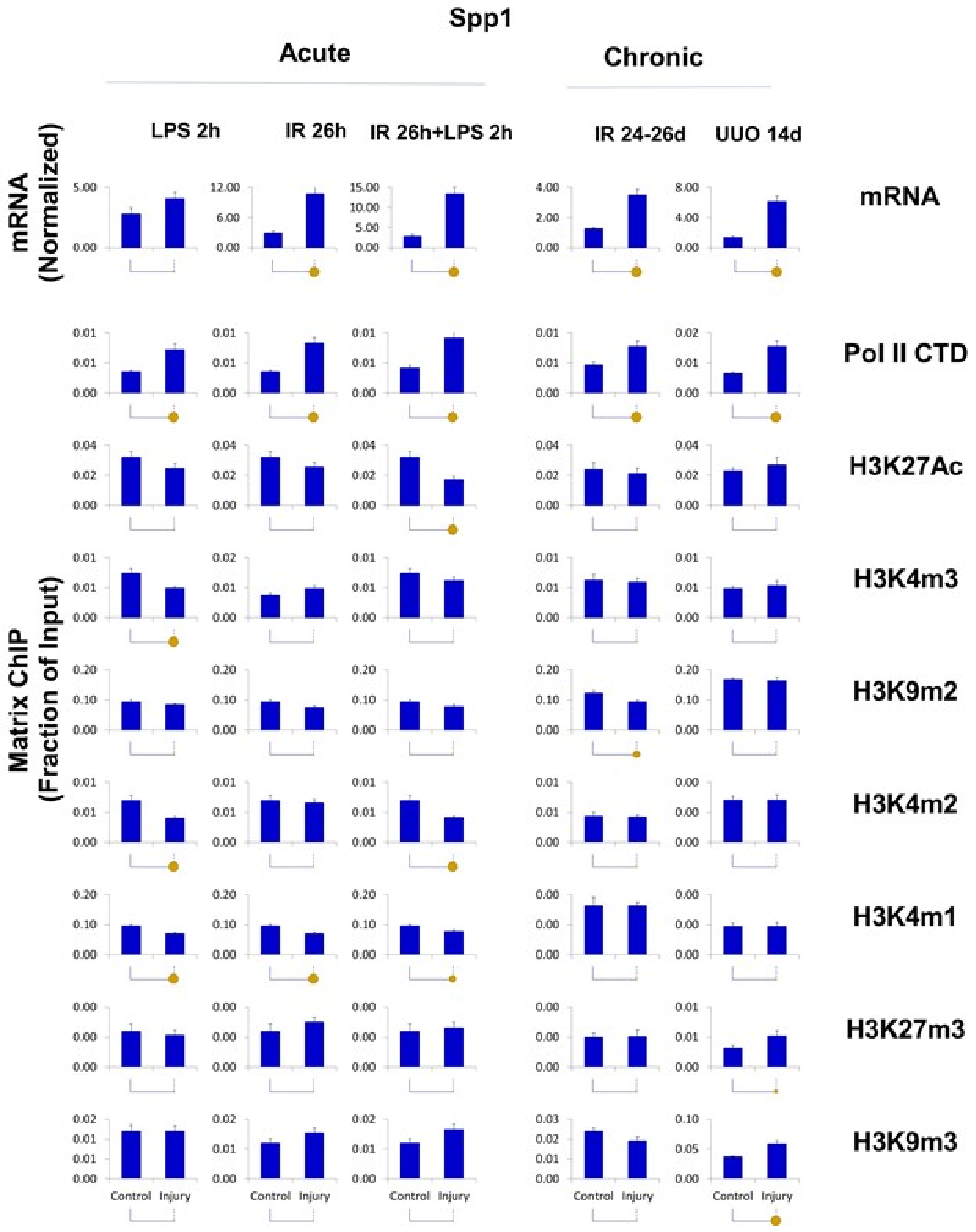

Fig.S7. Transcriptional and epigenetic analysis of Spp1 gene in AKI and CKD models. Transcript and ChIP assays and analysis were carried out as in Fig.S2 using primers to Spp1 (Tables S1-2). Data represents mean , n=6 mice from each group.

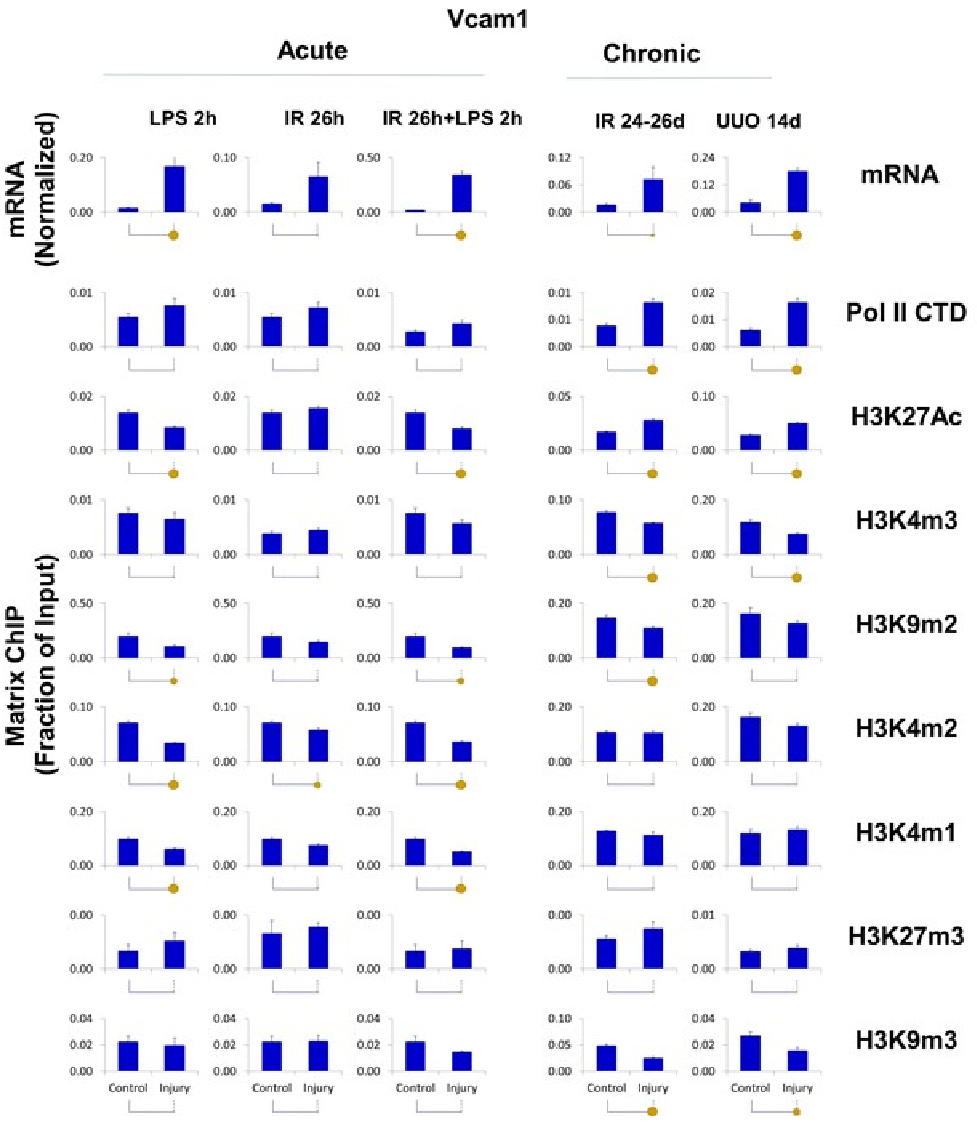

Fig.S8. Transcriptional and epigenetic analysis of Vcam1 gene in AKI and CKD models. Transcript and ChIP assays and analysis were carried out as in Fig.S2 using primers to Vcam1 (Tables S1-2). Data represents mean , n=6 mice from each group.

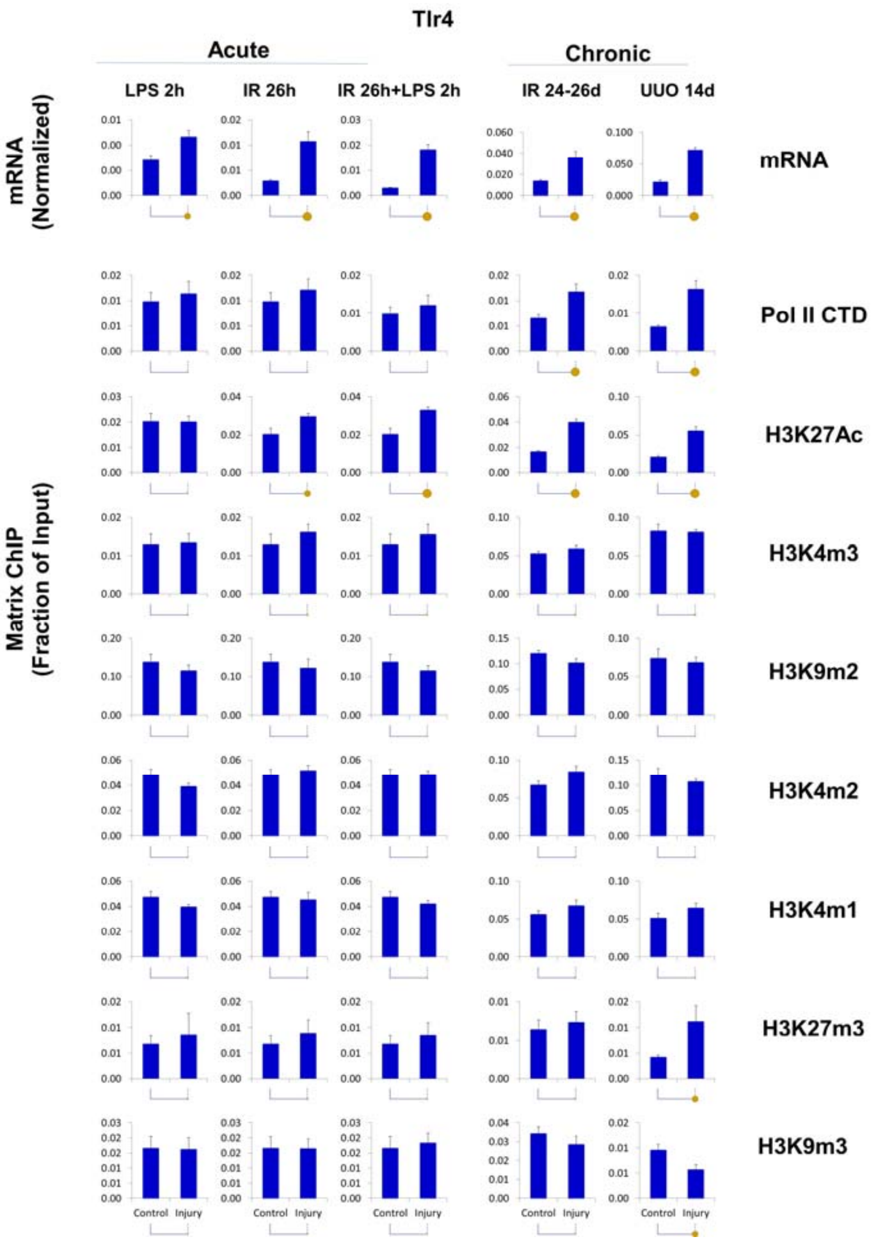

Fig.S9. Transcriptional and epigenetic analysis of Tlr4 gene in AKI and CKD models.

Transcript and ChIP assays and analysis were carried out as in Fig.S2 using primers to Trl4 (Tables S1-2). Data represents mean , n=6 mice from each group.

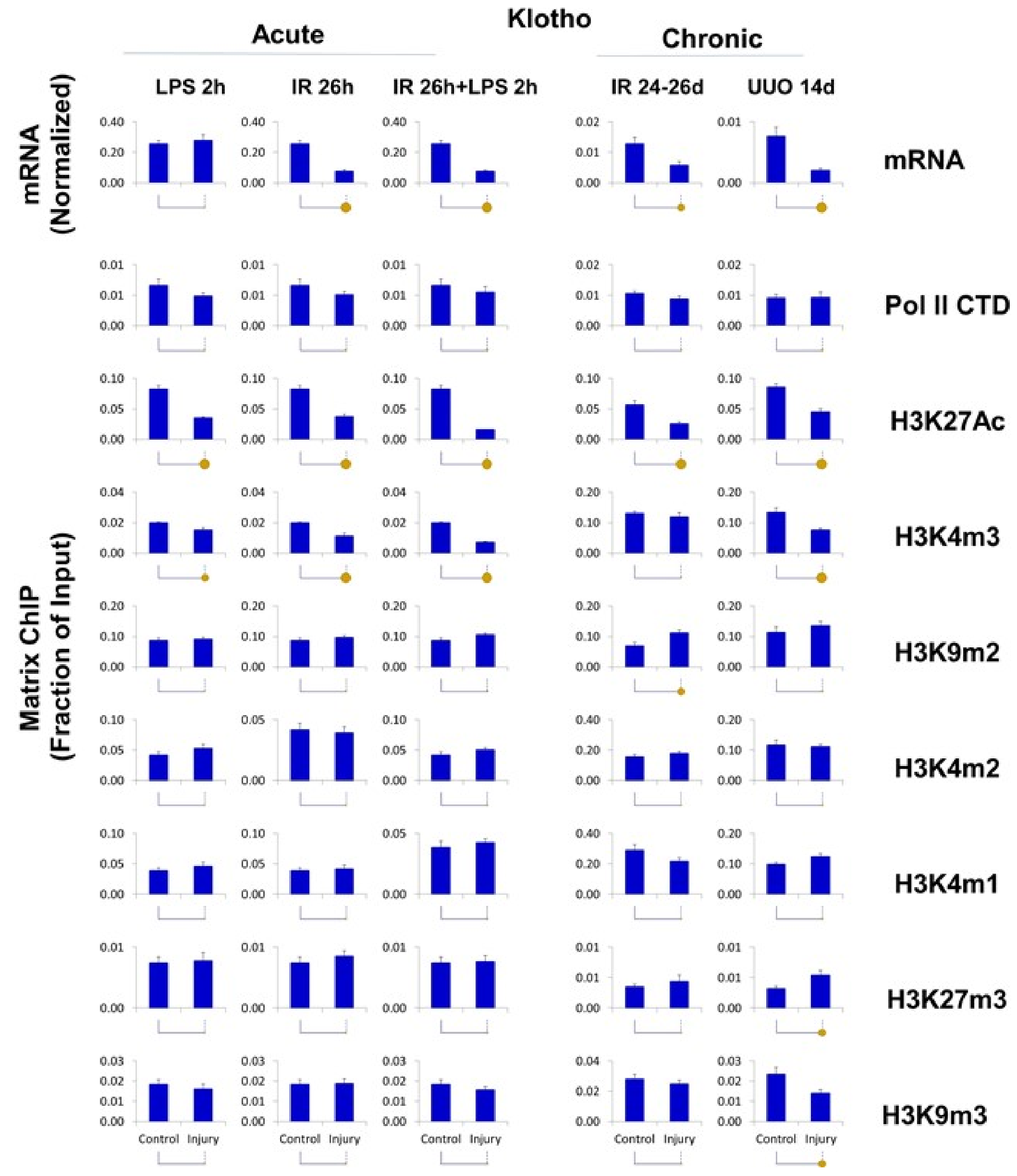

Fig.S10. Transcriptional and epigenetic analysis of Klotho gene in AKI and CKD models. Transcript and ChIP assays and analysis were carried out as in Fig.S2 using primers to Klotho (Tables S1-2). Data represents mean , n=6 mice from each group.

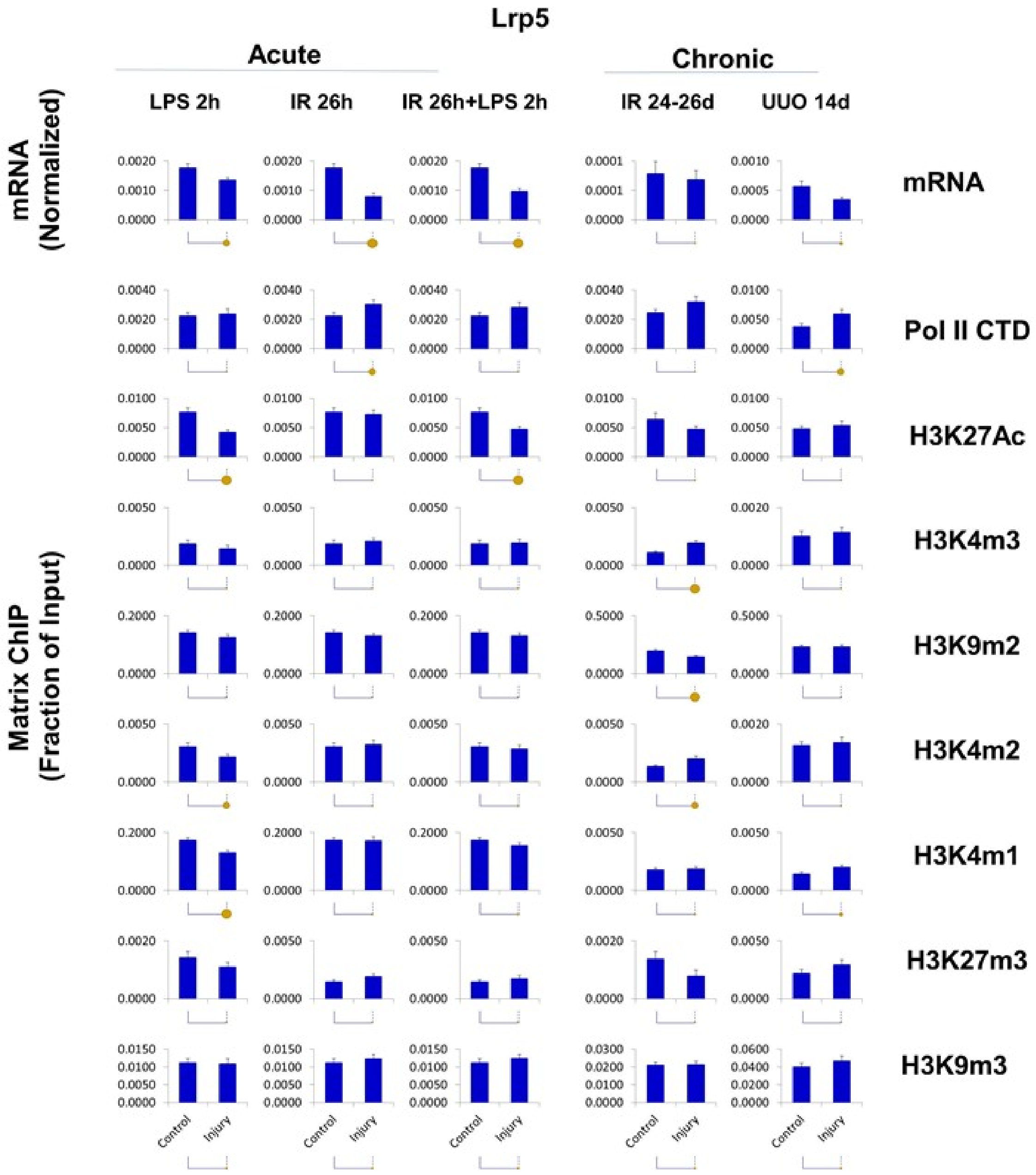

Fig.S11. Transcriptional and epigenetic analysis of Lrp5 gene in AKI and CKD models.

Transcript and ChIP assays and analysis were carried out as in Fig.S2 using primers to Lrp5 (Tables S1-2). Data represents mean , n=6 mice from each group.

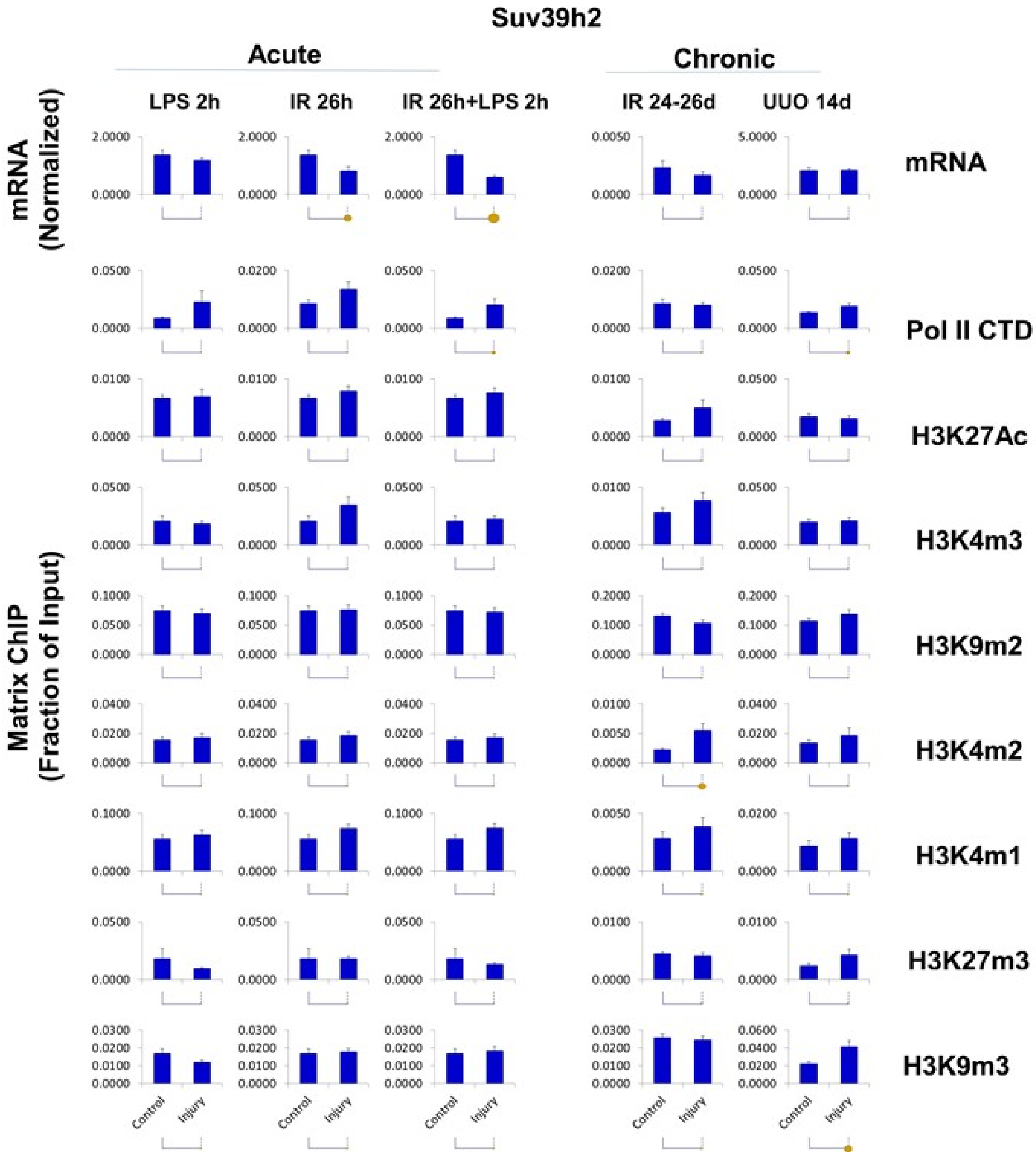

Fig.S12. Transcriptional and epigenetic analysis of Suv39h2 gene in AKI and CKD models.

Transcript and ChIP assays and analysis were carried out as in Fig.S2 using primers to Suv39h2 (Tables S1-2). Data represents mean , n=6 mice from each group.

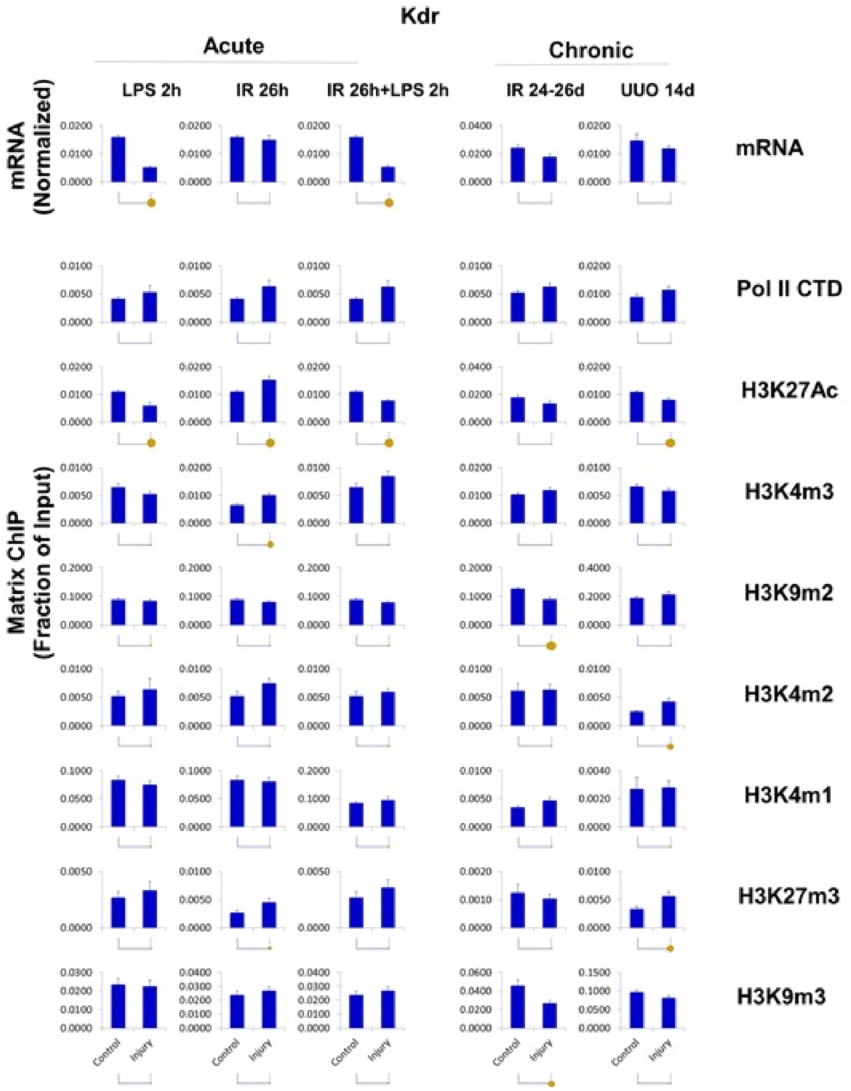

Fig.S13. Transcriptional and epigenetic analysis of Kdr gene in AKI and CKD models.

Transcript and ChIP assays and analysis were carried out as in Fig.S2 using primers to Kdr (Tables S1-2). Data represents mean , n=6 mice from each group.

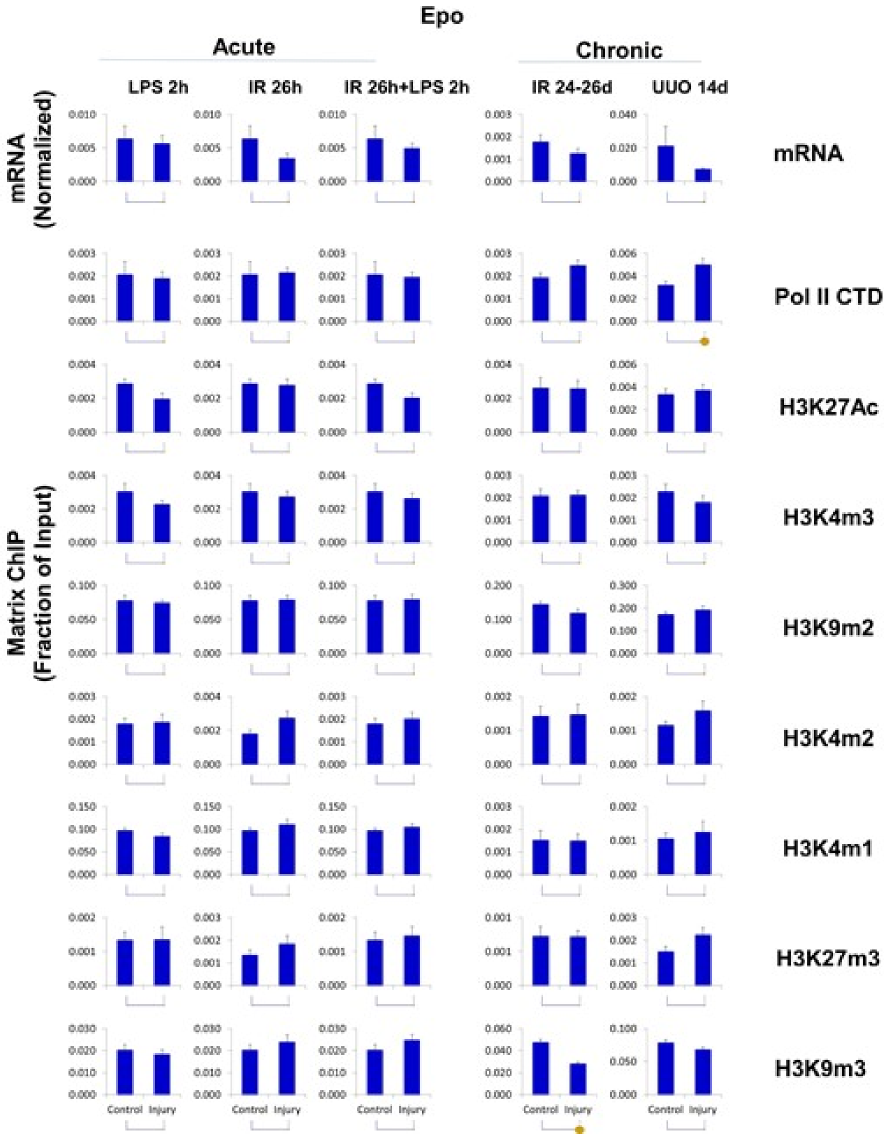

Fig.S14. Transcriptional and epigenetic analysis of Epo gene in AKI and CKD models.

Transcript and ChIP assays and analysis were carried out as in Fig.S2 using primers to Epo (Tables S1-2). Data represents mean , n=6 mice from each group.

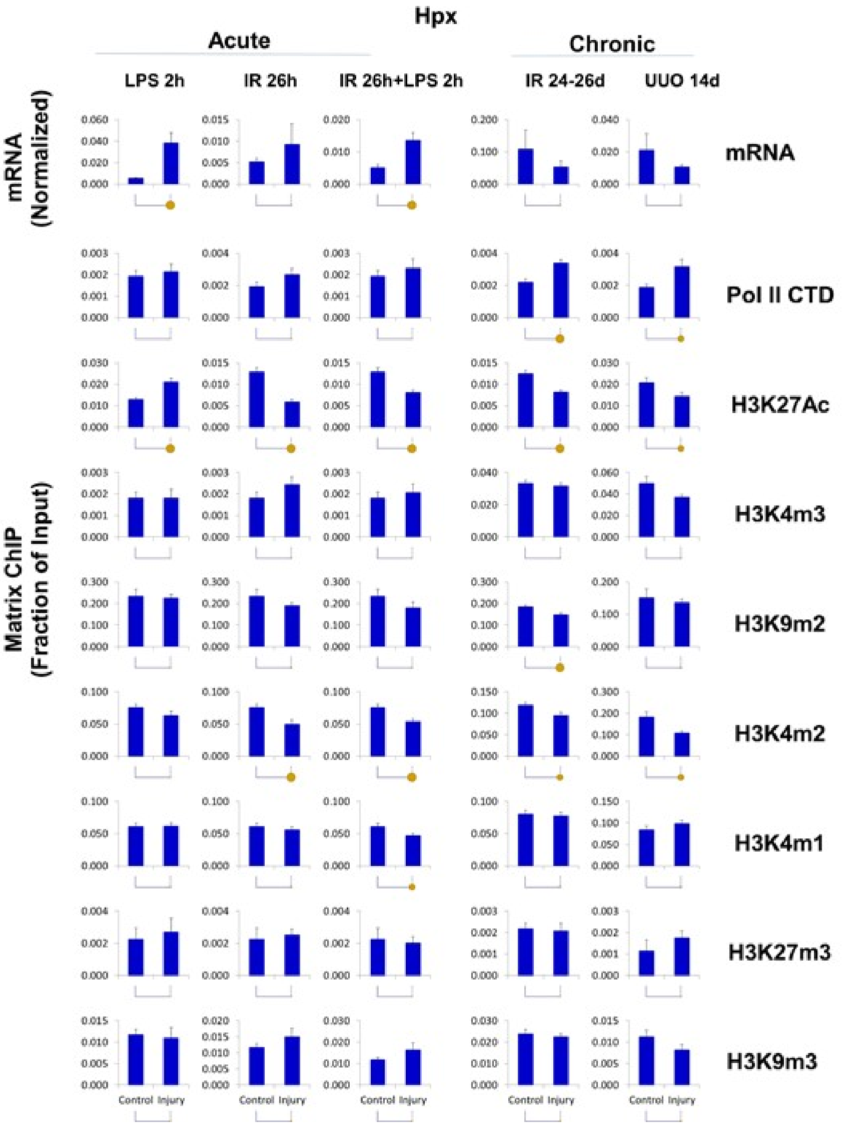

Fig.S15. Transcriptional and epigenetic analysis of Hpx gene in AKI and CKD models.

Transcript and ChIP assays and analysis were carried out as in Fig.S2 using primers to Hpx (Tables S1-2). Data represents mean , n=6 mice from each group.

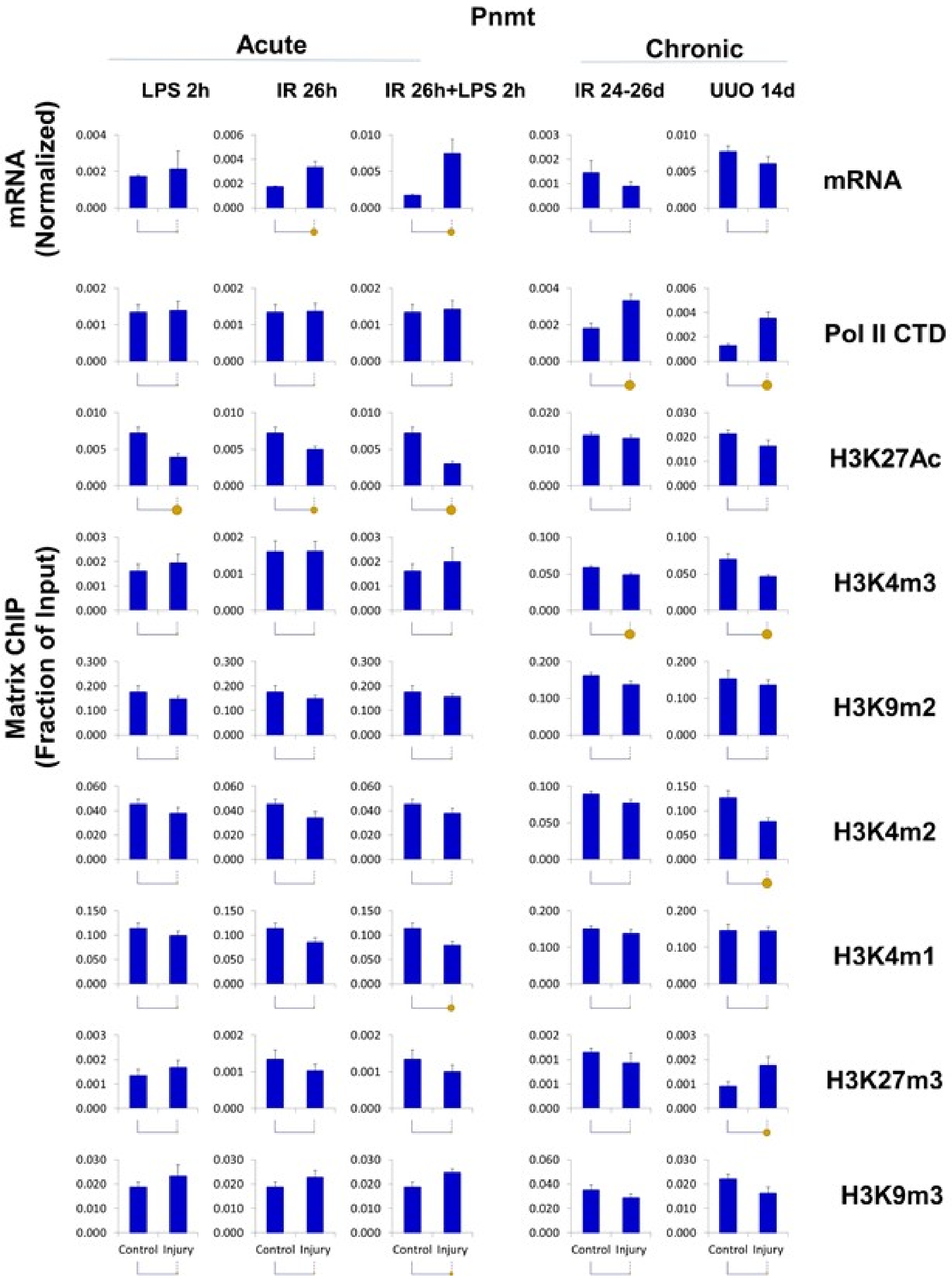

Fig.S16. Transcriptional and epigenetic analysis of Pnmt gene in AKI and CKD models.

Transcript and ChIP assays and analysis were carried out as in Fig.S2 using primers to Pnmt (Tables S1-2). Data represents mean , n=6 mice from each group.

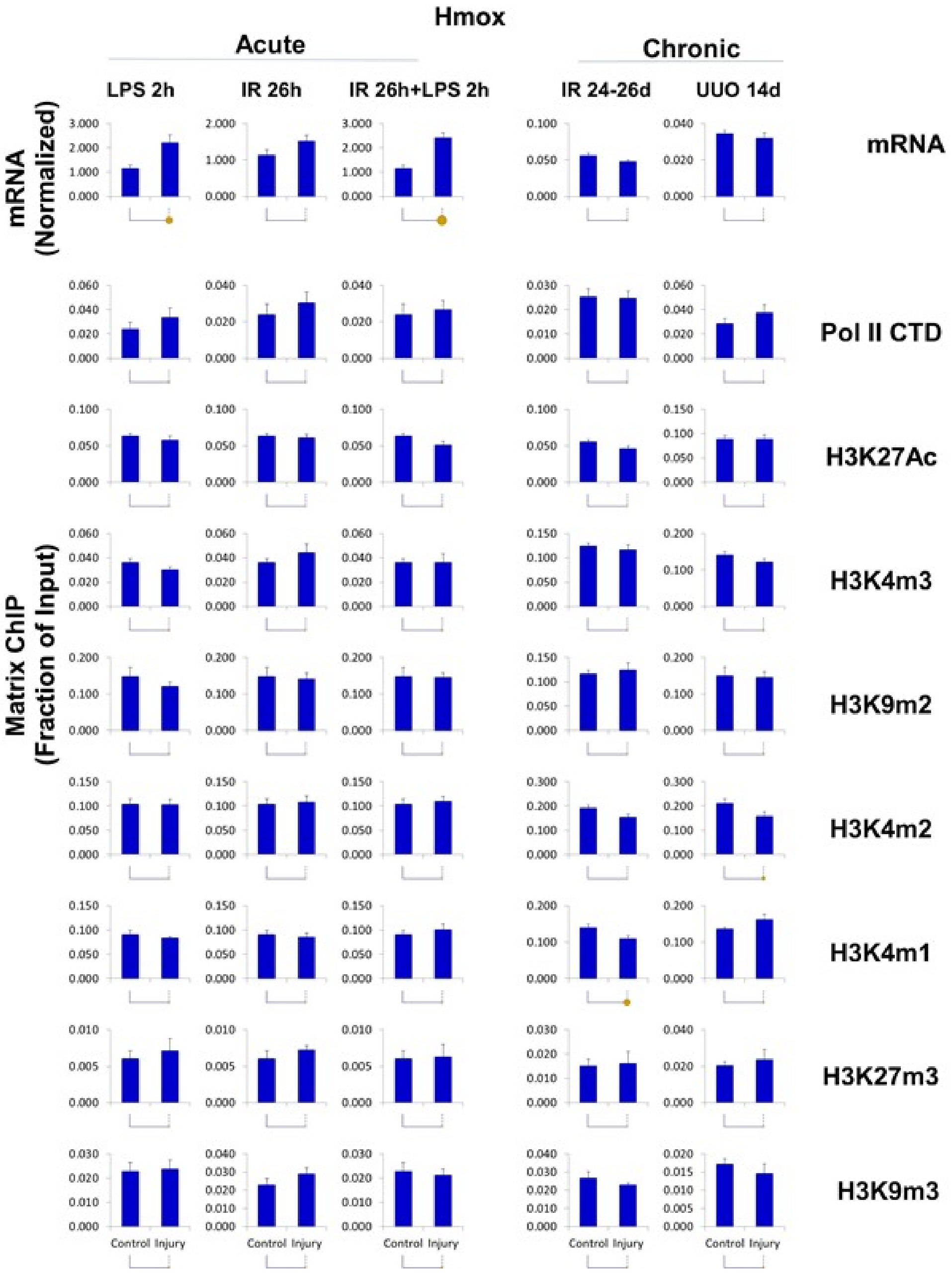

Fig.S17. Transcriptional and epigenetic analysis of Hmox gene in AKI and CKD models.

Transcript and ChIP assays and analysis were carried out as in Fig.S2 using primers to Hmox (Tables S1-2). Data represents mean , n=6 mice from each group.

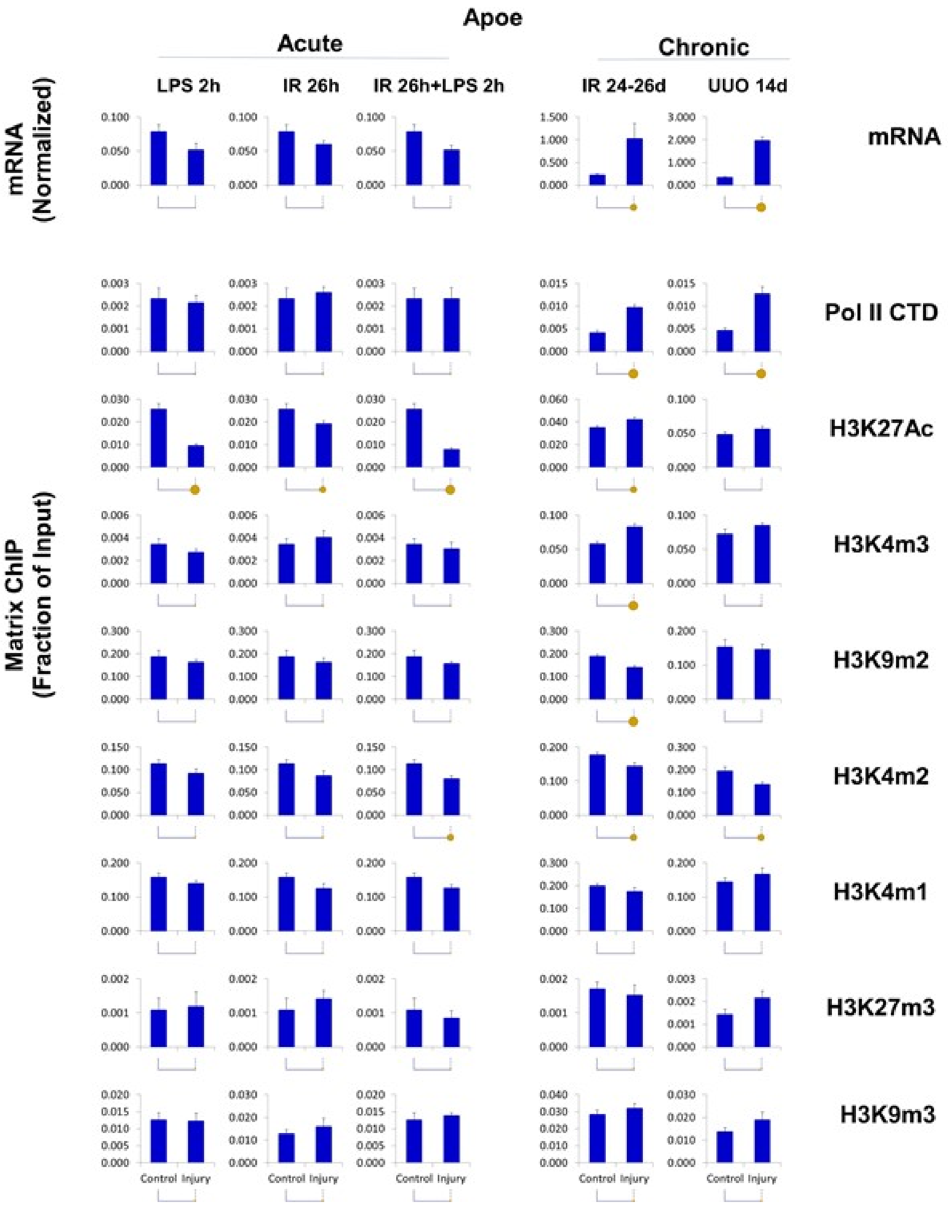

Fig.S18. Transcriptional and epigenetic analysis of Apoe gene in AKI and CKD models.

Transcript and ChIP assays and analysis were carried out as in Fig.S2 using primers to Apoe (Tables S1-2). Data represents mean , n=6 mice from each group.

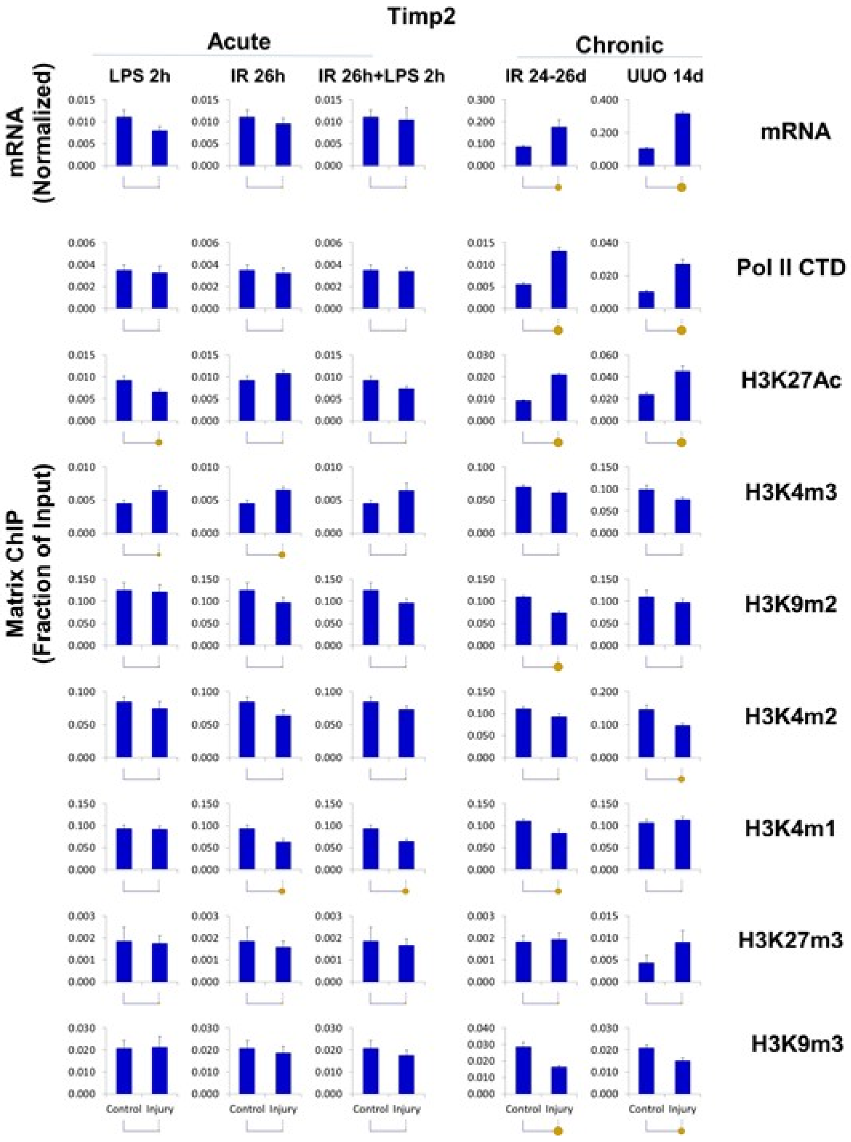

Fig.S19. Transcriptional and epigenetic analysis of Timp2 gene in AKI and CKD models.

Transcript and ChIP assays and analysis were carried out as in Fig.S2 using primers to Timp2 (Tables S1-2). Data represents mean , n=6 mice from each group.

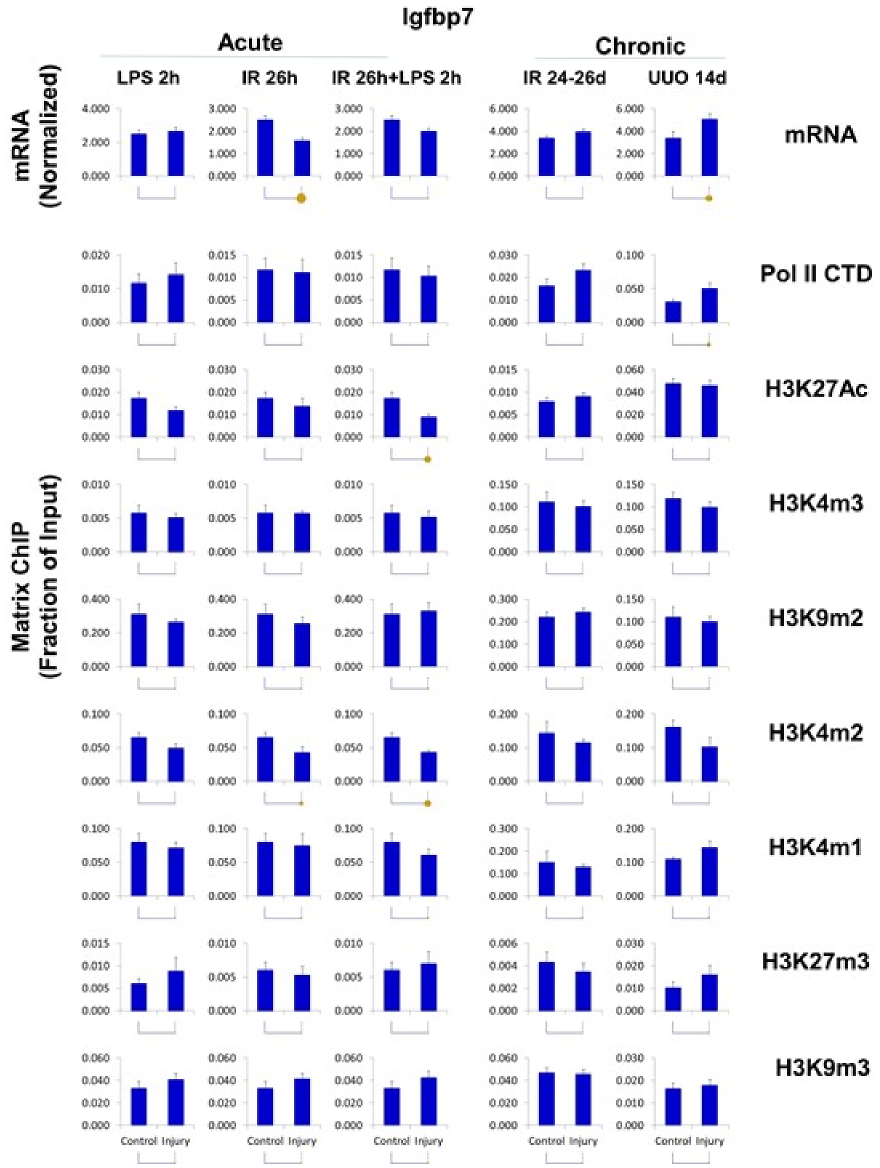

Fig.S20. Transcriptional and epigenetic analysis of Igfbp7 gene in AKI and CKD models.

Transcript and ChIP assays and analysis were carried out as in Fig.S2 using primers to Igfbp7 (Tables S1-2). Data represents mean , n=6 mice from each group.
